# Eicosapentaenoic acid potentiates the therapeutic effects of adipose tissue-derived mesenchymal stromal cells on lung and distal organ injury in experimental sepsis

**DOI:** 10.1186/s13287-019-1365-z

**Published:** 2019-08-23

**Authors:** Johnatas D. Silva, Miquéias Lopes-Pacheco, Ligia L. de Castro, Jamil Z. Kitoko, Stefano A. Trivelin, Natália R. Amorim, Vera L. Capelozzi, Marcelo M. Morales, Bianca Gutfilen, Sergio A. L. de Souza, Daniel J. Weiss, Bruno L. Diaz, Patricia R. M. Rocco

**Affiliations:** 1 Laboratory of Pulmonary Investigation, Carlos Chagas Filho Institute of Biophysics, Centro de Ciências da Saúde, Universidade Federal do Rio de Janeiro, Avenida Carlos Chagas Filho, s/n, Bloco G-014, Ilha do Fundão, Rio de Janeiro, RJ 21941-902 Brazil; 2 Laboratory of Inflammation, Carlos Chagas Filho Biophysics Institute, Federal University of Rio de Janeiro, Rio de Janeiro, Brazil; 3 Department of Pathology, School of Medicine, University of São Paulo, São Paulo, Brazil; 4 Laboratory of Cellular and Molecular Physiology, Carlos Chagas Filho Biophysics Institute, Federal University of Rio de Janeiro, Rio de Janeiro, Brazil; 5 National Institute of Science and Technology for Regenerative Medicine, Rio de Janeiro, Brazil; 6 Department of Radiology, School of Medicine, Federal University of Rio de Janeiro, Rio de Janeiro, Brazil; 7 Department of Medicine, College of Medicine, University of Vermont, Burlington, VT USA

**Keywords:** Sepsis, Mesenchymal stromal cells, Preconditioning, Eicosapentaenoic acid, Inflammation, Macrophages

## Abstract

**Background:**

Even though mesenchymal stromal cells (MSCs) mitigate lung and distal organ damage in experimental polymicrobial sepsis, mortality remains high. We investigated whether preconditioning with eicosapentaenoic acid (EPA) would potentiate MSC actions in experimental sepsis by further decreasing lung and distal organ injury, thereby improving survival.

**Methods:**

In C57BL/6 mice, sepsis was induced by cecal hligation and puncture (CLP); sham-operated animals were used as control. Twenty-four hours after surgery, CLP mice were further randomized to receive saline, adipose tissue-derived (AD)-MSCs (10^5^, nonpreconditioned), or AD-MSCs preconditioned with EPA for 6 h (10^5^, EPA-preconditioned MSCs) intravenously. After 24 h, survival rate, sepsis severity score, lung mechanics and histology, protein level of selected biomarkers in lung tissue, cellularity in blood, distal organ damage, and MSC distribution (by technetium-99m tagging) were analyzed. Additionally, the effects of EPA on the secretion of resolvin-D_1_ (RvD_1_), prostaglandin E_2_ (PGE_2_), interleukin (IL)-10, and transforming growth factor (TGF)-β1 by MSCs were evaluated in vitro.

**Results:**

Nonpreconditioned and EPA-preconditioned AD-MSCs exhibited similar viability and differentiation capacity, accumulated mainly in the lungs and kidneys following systemic administration. Compared to nonpreconditioned AD-MSCs, EPA-preconditioned AD-MSCs further reduced static lung elastance, alveolar collapse, interstitial edema, alveolar septal inflammation, collagen fiber content, neutrophil cell count as well as protein levels of interleukin-1β and keratinocyte chemoattractant in lung tissue, and morphological abnormalities in the heart (cardiac myocyte architecture), liver (hepatocyte disarrangement and Kupffer cell hyperplasia), kidney (acute tubular necrosis), spleen (increased number of megakaryocytes and lymphocytes), and small bowel (villi architecture disorganization). EPA preconditioning of MSCs resulted in increased secretion of pro-resolution and anti-inflammatory mediators (RvD_1_, PGE_2_, IL-10, and TGF-β).

**Conclusions:**

Compared to nonpreconditioned cells, EPA-preconditioned AD-MSCs yielded further reductions in the lung and distal organ injury, resulting in greater improvement in sepsis severity score and higher survival rate in CLP-induced experimental sepsis. This may be a promising therapeutic approach to improve outcome in septic patients.

**Electronic supplementary material:**

The online version of this article (10.1186/s13287-019-1365-z) contains supplementary material, which is available to authorized users.

## Background

Sepsis remains a major cause of morbidity and mortality worldwide, despite improvements in early diagnosis, antibiotic therapy, and clinical management. Several pharmacotherapeutic approaches have been tested but failed at improving outcomes in clinical trials, thus leaving an urgently unmet need for this devastating condition [1, 2].

Mesenchymal stromal cells (MSCs)—isolated from the bone marrow, adipose tissue, and other sources—are now known to induce immunomodulation following systemic administration, either by cell contact-dependent mechanisms or by secretion of paracrine factors, resulting in anti-inflammatory and reparative effects in a wide range of diseases [3–6]. In preclinical models of sepsis, MSC administration improved survival, but did not restore organ function [7–11]. Although no safety concerns have been observed in initial clinical trials, very few studies have been conducted in critically ill patients [12–14]. On the experimental front, recent studies have attempted to condition MSCs prior to administration in order to potentiate their therapeutic actions [6, 15–17].

Eicosapentaenoic acid (EPA), an omega-3 polyunsaturated fatty acid, may be an interesting agent for MSC preconditioning, since it has shown to modulate inflammatory and remodeling responses by altering profiling of secreted mediators in several inflammatory diseases [18, 19]. We previously found that EPA-exposed MSCs demonstrated enhanced secretion of anti-inflammatory and pro-resolution factors, such as resolvin D_1_, prostaglandin E_2_ (PGE_2_), interleukin (IL)-10, and transforming growth factor (TGF)-β. Additionally, EPA-MSCs were more potent than non-exposed MSCs in reducing lung inflammation and remodeling in a preclinical model of allergic asthma [17]. We hypothesized whether preconditioning of adipose tissue-derived (AD)-MSCs with EPA might enhance the therapeutic effects of AD-MSCs and thus mitigate lung and multiple organ injury caused by polymicrobial sepsis.

In this current study, the actions of nonpreconditioned and EPA-preconditioned AD-MSCs were comparatively investigated in a widely utilized murine model of sepsis: cecal ligation and puncture (CLP). Endpoint analyses included survival rate; sepsis severity score; lung mechanics and histology; protein levels of selected mediators in lung tissue; total and differential cell counts in blood; effects on morphological abnormalities in the liver, kidney, heart, spleen, and small bowel; macrophage capacity for phagocytosis; and AD-MSC distribution (by technetium-99m tagging).

## Methods

### Ethics statement

This study was approved by the Animal Care and Use Committee (CEUA:121/18) of the Health Sciences Center, Federal University of Rio de Janeiro, Rio de Janeiro, Brazil. All animals received humane care in compliance with the “Principles of Laboratory Animal Care” formulated by the National Society for Medical Research and the US National Academy of Sciences *Guide for the Care and Use of Laboratory Animals*. Animals were housed in standard laboratory cages (12-h light/dark cycles, temperature 23 ± 1 °C), three to a cage, with access to food and water ad libitum.

### Animal preparation and experimental protocol

All experimental assessments were performed in blind fashion. A total of 152 C57BL/6 mice (144 females and 8 males, weight 25–30 g, age 8–10 weeks), obtained from the animal care facility of the Laboratory of Pulmonary Investigation, Federal University of Rio de Janeiro, were used: 32 females for lung mechanics, histology, biomarker measurement, and distal organ analysis; 32 females for the analysis of blood cellularity; 40 females for the analysis of clinical score and survival rate; 20 females for the analysis of biodistribution in tissues; 20 females to obtain alveolar macrophages; and 8 males as cell donors. All animals were randomly allocated across two groups: control and sepsis (Additional file 1: Figure S1). Experimental sepsis was induced using an established model of cecal ligation and puncture (CLP) surgery, as described elsewhere [20]. Briefly, animals were anesthetized by intraperitoneal (i.p.) injection of ketamine and xylazine (0.25 mg kg^−1^ and 0.025 mg kg^−1^, respectively) and a laparotomy (2-cm incision) was performed. The cecum was carefully isolated to prevent damage and ligated with a 3-0 cotton suture. The ligature was placed below the ileocecal valve to prevent bowel obstruction. The cecum was punctured twice with an 18-gauge needle and the animals left to recover from anesthesia. In the control group (C), a sham surgery was performed, in which the abdominal cavity was opened, and the cecum was isolated, but without ligation and puncture. Postoperative care was similar in both groups, consisting of a subcutaneous injection of tramadol hydrochloride (20 mg g^−1^ body weight) in 1 mL warm (37 °C) normal saline (NaCl 0.9%). Twenty-four hours after surgery, the CLP group was subsequently randomized into three subgroups to receive sterile saline (0.05 mL, SAL), nonpreconditioned AD-MSCs (1 × 10^5^ cells per mouse, concentrated in 0.05 mL of sterile saline, AD-MSC), or eicosapentaenoic acid (EPA)-preconditioned AD-MSCs (1 × 10^5^ cells per mouse, concentrated in 0.05 mL of sterile saline, AD-MSC-EPA) via the intrajugular vein. Twenty-four hours after therapy, mice were euthanized and data analyzed, except for those mice used to evaluate the survival rate, which were euthanized on day 7.

### Adipose tissue MSC isolation, characterization, and preconditioning

Male C57BL/6 mice (weight 20–25 g, age 8–10 weeks) were anesthetized with intravenous ketamine (25 mg/kg) and xylazine (2 mg/kg) and used as cell donors. Adipose tissue was obtained from the epididymal fat pads as described elsewhere [3]. Tissues were collected, rinsed in PBS, transferred to a Petri dish, and cut into small pieces. The dissected pieces were washed with PBS and subsequently digested with type I collagenase (1 mg mL^−1^ in PBS) for 30–40 min at 37 °C. After digestion, fresh medium was added, and the suspension was centrifuged at 400×*g* for 10 min at room temperature. The pellets were resuspended in DMEM containing 1% antibiotic solution (Invitrogen, CA, USA), 20% FBS, and 15 mM HEPES; seeded in T25 flasks (4 mL per flask); and incubated at 37 °C in a humidified atmosphere containing 5% CO_2_. On day 3 of culture, the medium was replaced, and non-adherent cells were removed. Adherent cells reaching 80% confluence were passaged with 0.25% trypsin-EDTA solution (Gibco, NM, USA). Cells from the third passage were characterized on the basis of the following criteria: (1) MSCs must be plastic-adherent when maintained in standard culture conditions using tissue culture flasks and (2) 95% of the MSC population must express specific surface antigens by flow cytometry [3]. AD-MSCs were preconditioned or not with EPA (10 μM, CAS 10417-94-4, Cayman Chemical, Ann Arbor, MI) for 6 h. For therapeutic injection, cells were detached with trypsin, washed, and resuspended in sterile saline.

Flow cytometry was performed using commercially available antibodies against CD45 (hematopoietic marker), CD31 (endothelial cell marker), MHC class II, CD29 (β1-integrin), CD49e (integrin alpha-5), and CD44 (hyaluronic acid receptor), all from BD Biosciences (São Paulo, Brazil).

Additionally, cell survival and viability were investigated by using annexin V-FITC and propidium iodide (PI) staining [6]. Briefly, harvested AD-MSCs were resuspended in 1× binding buffer containing annexin V-FITC (Calbiochem, Billerica, MA). After incubation for 15 min at room temperature, cell suspension was diluted with 1× binding buffer and incubated with PI. After 15 min at room temperature, cells were subjected to flow cytometry acquisition. All data were acquired in a FACSCalibur flow cytometer (BD Biosciences Immunocytometry Systems, San Jose, CA) and analyzed using Flow Jo X 10.0.7 software (Tree Star Inc., Ashland, OR).

To collect extracellular vesicles (EVs), the cells were cultured with serum-free medium for 48 h. The medium was collected and centrifuged at 2000×*g* for 20 min at 4 °C to remove cellular debris, followed by two rounds of ultracentrifugation (100,000×*g*) for 1 h each at 4 °C. The precipitate was collected and suspended in 0.9% saline solution for immediate use. Following collection of conditioned media for EV isolation, nonpreconditioned or EPA-preconditioned AD-MSCs were fixed in 2.5% glutaraldehyde in 0.1 M sodium cacodylate buffer (pH 7.2) for 2 h and washed twice with cacodylate buffer. Immediately thereafter, post-fixation with OsO_4_ and FeCNK solution (1:1) was performed for 45 min, followed by dehydration in a graded ethanol series for 10 min at each concentration (30%, 50%, 70%, 90%, 100%, the latter three times). After critical point drying, the coverslips were analyzed and ultrastructural images of EVs being released from AD-MSC surfaces were acquired in a FEI QUANTA 250 scanning electron microscope (FEI, USA). The size distribution and concentration of EVs were evaluated by dynamic light scattering (DLS) using a Zetasizer Nano ZS ZEN3600 (Malvern Instruments, Malvern, UK) equipped with a solid-state He-Ne laser at 633-nm wavelength. The intensity of the scattered light was measured at 173°. All measurements were undertaken in triplicates at 25 °C. Data processing and analysis were performed using Zetasizer software version 7.03.

### Isolation of alveolar macrophages and phagocytosis capacity

Alveolar macrophages were obtained from the bronchoalveolar lavage fluid (BALF) of C, CLP-SAL, CLP-AD-MSC, and CLP-AD-MSC-EPA mice [17, 21]. Experiments were performed in triplicate. BALF was centrifuged at 300*g* for 10 min and the cellular *pellet* was washed with saline, resuspended in red blood cell lysis buffer (8.3 g NH_4_Cl, 1 g KHCO_3_, 1.8 mL 5% EDTA in 1 L distilled water) for 5 min at room temperature, and centrifuged again at 300*g* for 10 min. The pelleted cells were resuspended and cultured in a 12-well culture plate at 37 °C with 5% CO_2_ at a concentration of 10^5^ cells per well in 1 mL RPMI 1640 medium (Sigma Chemical Co., St. Louis, MO) supplemented with 10% FBS, 1 mM pyruvate, 1% nonessential amino acids, 14 mM glucose, 17.9 mM NaHCO_3_, 10 mM HEPES, 100 U/mL penicillin, and 0.1 mg/mL streptomycin. After 2 h of incubation, non-adherent cells were washed off with saline, and the medium was refreshed. Alveolar macrophages were stimulated with conditioned media obtained from AD-MSCs stimulated or not with EPA for an additional 24 h. Alveolar macrophages were then washed with sterile saline, harvested from the culture plates, and pelleted by centrifugation (600*g* for 5 min).

The phagocytosis capacity of alveolar macrophages was tested with pHrodo™ Green Zymosan A BioParticles® (Life Technologies, Carlsbad, USA), in accordance with the supplier’s instructions. Briefly, alveolar macrophages were plated with nonpreconditioned or EPA-preconditioned AD-MSCs on a 96-well tissue culture plate and incubated in RPMI1640 10% FBS with 1% penicillin/streptomycin for 2 h at 37 °C, in a 5% CO_2_ atmosphere, for cell adherence. Wells were then washed with saline solution, and alveolar macrophages incubated with media containing fluorescently labeled zymosan particles (0.5 mg mL^−1^) for 2 h. Fluorescence was measured in a microplate reader (Perkin-Elmer, Waltham, USA) [18]. Phagocytosis of fluorescently labeled *Saccharomyces cerevisiae* BioParticles was quantified by measuring intracellular fluorescence emitted by engulfed particles at 585 nm.

### Clinical sepsis severity score and survival rate

At 24 h (time therapy started) and 48 h (time of analysis) after CLP surgery, a cohort of mice had their clinical score evaluated. The score consisted of analyzing the following parameters: presence of piloerection, altered respiration rate, fecal alterations, lacrimation/eyelid changes, contraction of the abdomen, lack of strength when grasping, change in body temperature, escape response after touch, exploration of the environment, and compromised locomotor activity. For the presence of each of the aforementioned parameters, one point was computed, while no point was given in the absence of such abnormalities. The score was then added for each mouse and graded as follows: 0, no clinical abnormalities; 1 to 3, mild sepsis; 4 to 7, moderate sepsis; 8 to 10, severe sepsis. All mice had free access to water and food and were monitored for 7 days to evaluate survival rate. During this period, all animals received tramadol hydrochloride (20 mg g^−1^ body weight) via subcutaneous injection every 6 h for pain management.

### Lung mechanics

Twenty-four hours after AD-MSC administration, another set of animals were sedated (diazepam 1 mg kg^−1^ intraperitoneally), anesthetized (thiopental sodium 20 mg kg^−1^ intraperitoneally), tracheotomized, paralyzed (vecuronium bromide, 0.005 mg kg^−1^ intravenously), and ventilated using a constant-flow ventilator (Samay VR15; Universidad de la Republica, Montevideo, Uruguay) with the following settings: respiratory rate 100 breaths/min, tidal volume (*V*_T_) 0.2 mL, and fraction of inspired oxygen (FIO_2_) 0.21. The anterior chest wall was surgically removed and a positive end-expiratory pressure (PEEP) of 2 cmH_2_O applied. Airflow and tracheal pressure (Ptr) were measured. Lung mechanics were analyzed by the end-inflation occlusion method [22]. In an open-chest preparation, Ptr reflects transpulmonary pressure (PL). Briefly, after end-inspiratory occlusion, there is an initial rapid decline in PL from the preocclusion value down to an inflection point, followed by a slow pressure decay until a plateau is reached. This plateau corresponds to the elastic recoil pressure of the lung (Pel). Static lung elastance (Est,L) was determined by dividing Pel by *V*_T_. Lung mechanics measurements were performed ten times in each animal. All data were analyzed using ANADAT software (RHT-InfoData, Inc., Montreal, Quebec, Canada).

### Lung histology

The right lung was fixed in 4% buffered formalin and embedded in paraffin. Sections (4 μm thick) were stained with hematoxylin-eosin. Photomicrographs at magnifications of × 25, × 100, and × 400 were obtained from ten non-overlapping fields of view per section using a light microscope (Olympus BX51, Olympus Latin America Inc., Brazil). Diffuse alveolar damage (DAD) was quantified using a weighted scoring system by two investigators (J.D.S. and V.L.C.) blinded to group assignment. Briefly, scores of 0 to 4 were used to represent alveolar collapse, interstitial edema, and alveolar septal inflammation, with 0 standing for no effect and 4 for maximum severity. Additionally, the extent of each scored characteristic per field of view was determined on a scale of 0 to 4, with 0 standing for no visible evidence and 4 for complete involvement. Scores were calculated as the product of severity and extent of each feature, on a range of 0 to 16. The cumulative DAD score was calculated as the sum of each score characteristic and ranged from 0 to 48. The fraction area of total and differential cell counts in lung tissue was determined by the point-counting technique across ten random, non-overlapping microscopic fields at × 1000 magnification [23]. Collagen fibers (Picrosirius-polarization method) were quantified in alveolar septa at × 400 magnification. The area occupied by fibers was determined by digital densitometric recognition (Image-Pro Plus 7.1 software, Media Cybernetics, Silver Spring, MD, USA) and divided by the area of each septum [24].

### Total and differential cellularity in blood

Blood was collected with a heparinized needle through the abdominal vein. Total leukocyte numbers were measured in a Neubauer chamber under light microscopy after diluting the samples in Türk solution (2% acetic acid). Differential cell counts (monocytes and neutrophils, × 1000 magnification) were performed in Cytospin™ smears stained by the May–Grünwald–Giemsa method under a light microscope (Olympus BX51, Olympus Latin America Inc., Brazil).

### Pathologic findings in distal organs

The liver, kidney, heart, spleen, and small bowel were fixed in 4% buffered formalin, embedded in paraffin, and cut into slices 4 μm thick, which were stained with hematoxylin and eosin (Vetec Química Fina, Rio de Janeiro, Brazil). A semiquantitative scoring system based on severity and extension of injury was used to assess the degree of distal organs damage. Severity was graded as follows: 1 = normal tissue, 2 = mild injury, 3 = moderate injury, and 4 = severe injury. Extension of injury was graded as follows: 0 = normal tissue, 1 = damage to 1–25% of total tissue examined, 2 = damage to 26–50% of total tissue examined, 3 = damage to 51–75% of total tissue examined, and 4 = damage to 76–100% of total tissue examined. The final score is obtained by multiplying severity and extension of injury in each examined tissue. These analyses were performed by two expert pathologists (J.D.S. and V.L.C.).

### Analysis of multiple soluble factors in vivo and after in vitro stimulation using enzyme-linked immunosorbent assay (ELISA) and automated enzyme immunoassay (EIA)

For protein isolation, the right lobes of the lungs were frozen in liquid nitrogen and stored at − 80 °C until analysis. Lung tissue was homogenized in lysis buffer (PBS 1×, Triton X 0.01%, and 1× Roche protease inhibitor cocktail; Roche Diagnostic, Mannheim, Germany) using a glass Potter homogenizer with Teflon piston. Total cytokines were quantified in accordance with the manufacturer’s protocol and normalized to the total content of protein as quantified by Bradford’s reagent (Sigma-Aldrich, St Louis, MO, USA). Protein levels of interleukin (IL)-1β, KC (murine IL-8 homolog), IL-10, transforming growth factor (TGF)-β, and vascular endothelial growth factor (VEGF) (PeproTech, Rocky Hill, NJ, USA; R&D, Minneapolis, MN, USA) were quantified in lung homogenate with ELISA kits, in accordance with the manufacturers’ instructions.

Moreover, levels of IL-10, TGF-β, RvD1, and PGE_2_ were quantified in the supernatants of nonpreconditioned and EPA-preconditioned AD-MSCs. For this purpose, in vitro experiments were performed: 10^5^ AD-MSCs were added to a 12-well plate and cultured for 24 h under normal conditions (DMEM–high glucose medium supplemented with 10% FBS, 1000 U/mL penicillin/streptomycin, and 2 mM l-glutamine; Invitrogen Life Technologies, Grand Island, NY, USA). The next day, AD-MSCs received conditioned medium (FBS-free) and were stimulated or not with EPA for 6 h. After stimulation, the supernatants were collected and levels of IL-10, TGF-β (assessed by ELISA, Biolegend, San Diego, CA, USA), RvD1, and PGE_2_ (assessed by EIA, Cayman Chemical, Ann Arbor, MI, USA) were analyzed, as per manufacturer instructions. Results are expressed as picogram per milliliter.

### Biodistribution of AD-MSCs labeled with technetium-99m

Nonpreconditioned and EPA-preconditioned AD-MSCs were labeled with ^99m^TcO_4_^−^ as previously described [25]. All the procedures for cell preparation and labeling were carried out in a laminar flow hood. Briefly, 0.5 mL of fresh and sterile SnCl_2_ solution was added to the cell suspension in saline solution, and the mixture was incubated at room temperature for 10 min. Then, 45 MBq of ^99m^TcO_4_^−^ was added, and the incubation continued for another 10 min. After centrifugation (500×*g* for 5 min), the supernatant was removed, and the cells were washed once more with NaCl 0.9% solution. The pellet was suspended in NaCl 0.9%, and the viability of the labeled cells was assessed by the trypan blue exclusion test. Labeling efficiency (percentage) was calculated as: [(radioactivity of cell pellet)/(radioactivity of cell pellet + supernatant)] × 100.

### Statistical analysis

Sample size calculation was based on our previous experience using this CLP model [20]. The Kolmogorov–Smirnov test with Lilliefors’ corrections was used to test for normality of data, while the Levene median test was used to evaluate the homogeneity of variances. Parametric data were assessed using one-way analysis of variance (ANOVA) followed by Tukey’s test. Survival curves were derived by the Kaplan–Meier method and compared by the log-rank test. Nonparametric data were analyzed using the Kruskal–Wallis test followed by Dunn’s test. Parametric data were expressed as mean ± standard deviation (SD), while nonparametric data were expressed as median (interquartile range). All tests were performed in GraphPad Prism v6.01 (GraphPad Software, La Jolla, California, USA). A two-sided *P* value < 0.05 was considered significant.

## Results

### EPA preconditioning did not affect AD-MSC morphology, cell surface markers, survival, or expression of extracellular vesicles, but increased secretion of biomarkers by AD-MSCs

To evaluate whether EPA has any effect on AD-MSC, an in vitro analysis of AD-MSC morphology, cell surface markers, expression of extracellular vesicles, and biomarkers secreted by AD-MSCs was performed.

**Fig. 1:**
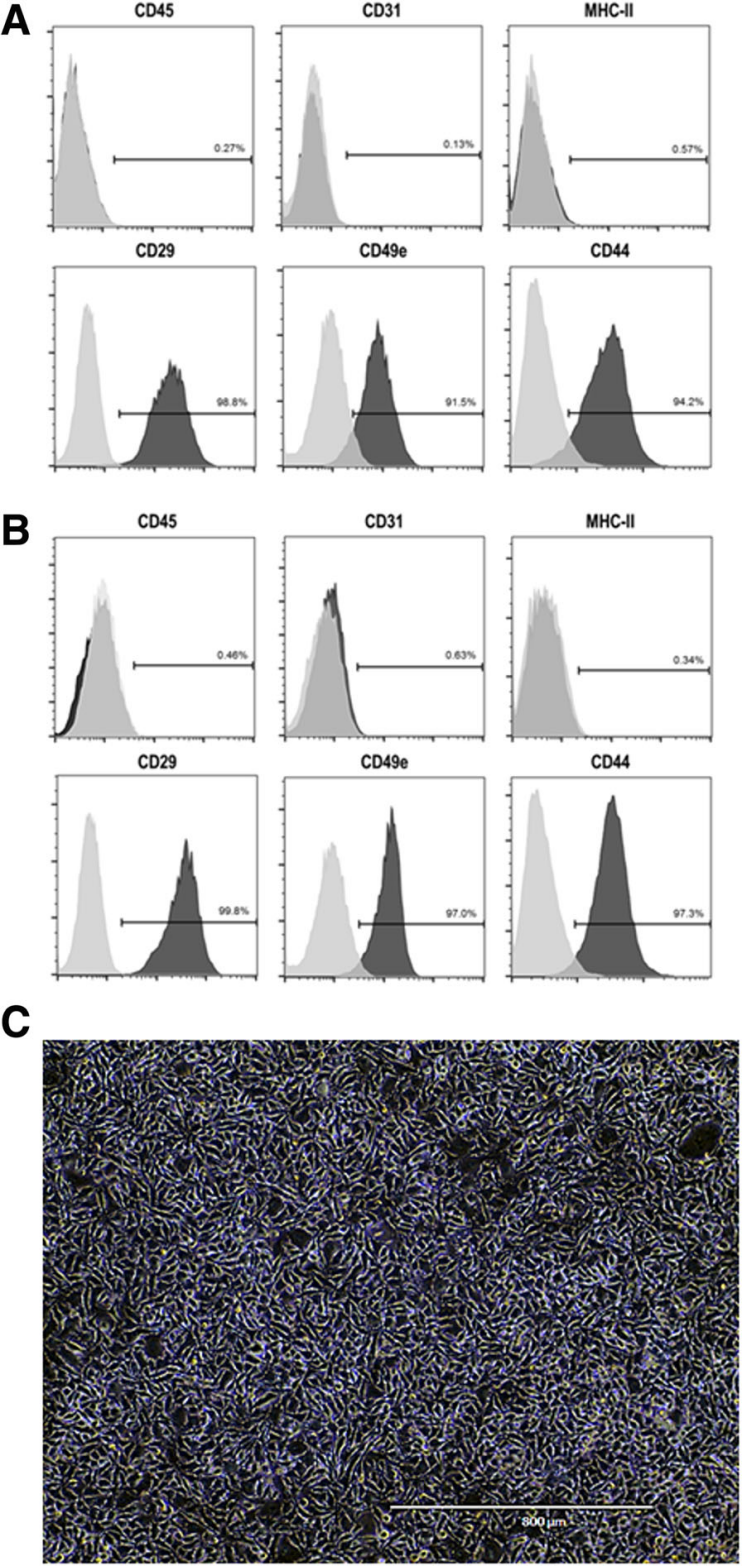
Characterization of adipose tissue (AD)-derived MSCs by flow cytometry. Nonpreconditioned (**a**) and EPA-preconditioned (**b**) AD-MSCs similarly demonstrated lack of expression of CD45, CD31, and MHCII, but expressed CD29, CD49e, and CD44, as determined by flow cytometry. Isotype controls were used as negative control (light gray histograms). *Y*-axis: cell count; *X*-axis: fluorescence intensity. **c** Note the homogeneous fibroblastoid morphology of adherent AD-MSCs, which is similar in nonpreconditioned (AD-MSC) and EPA-preconditioned AD-MSCs

**Fig. 2:**
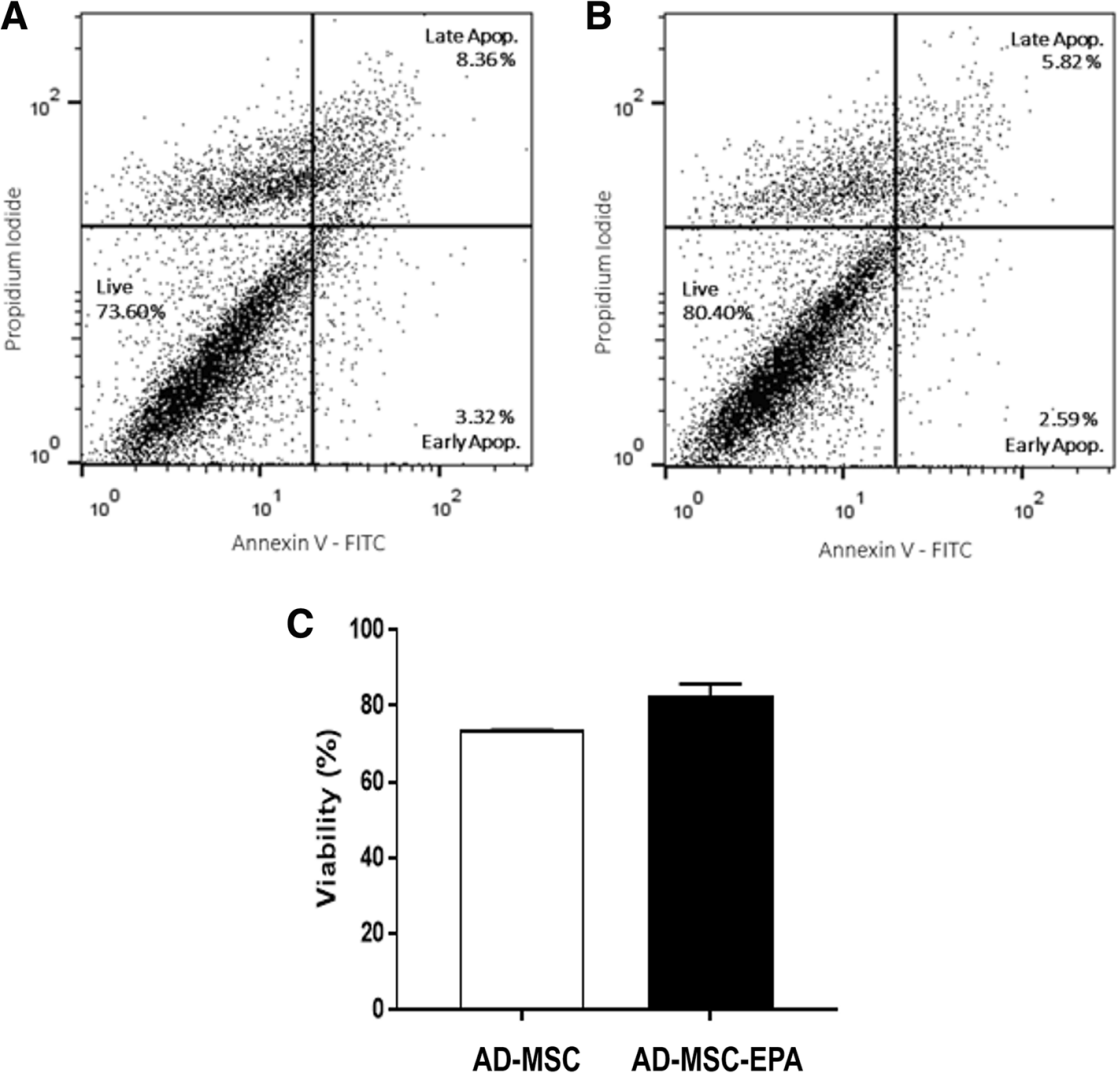
MSC viability. Viability was analyzed in nonpreconditioned (**a**) and EPA-preconditioned AD-MSCs (**b**). In **c**, bars represent viability quantification. Cells were stained with annexin V-FITC antibody and propidium iodide to assess the percentage of viable cells (annexin V/PI-cells) by flow cytometry

**Fig. 3:**
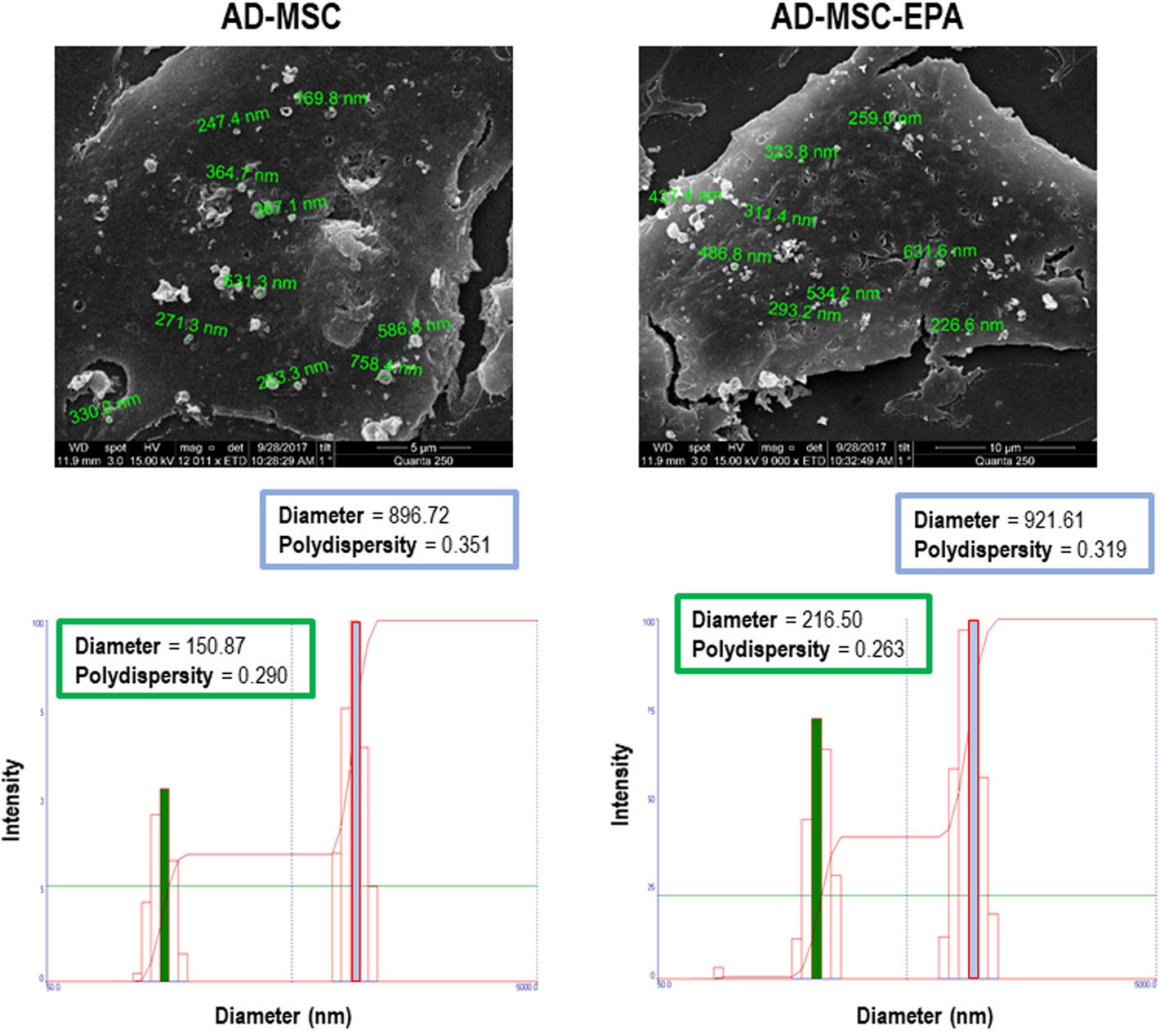
Upper panels: Scanning electron microscopy of adipose tissue (AD)-derived mesenchymal stromal cells (MSCs). Note the presence of extracellular vesicles on AD-MSC surfaces. Lower panels: Representative graph of the intensity and hydrodynamic diameter of extracellular vesicle samples, analyzed using the dynamic light scattering technique. Graph shows two populations of extracellular vesicles obtained from nonpreconditioned (AD-MSC) and EPA-preconditioned MSCs (AD-MSC-EPA): one of lower intensity and medium size, characteristic of exosomes, and another with greater intensity and average size, characteristic of microvesicles. Green and blue lines denote the mean diameter

**Fig. 4:**
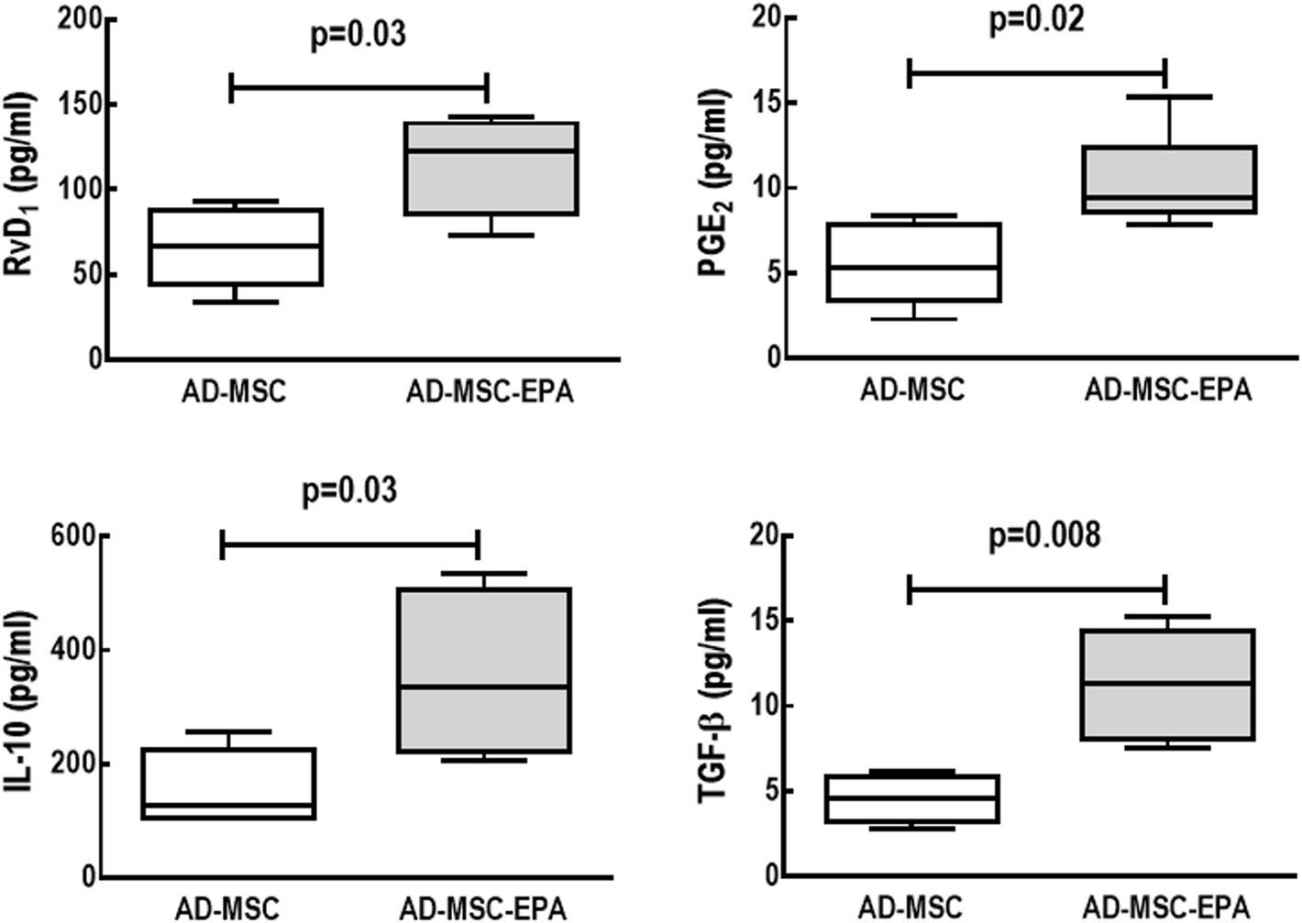
Levels of resolvin (RvD_1_) and prostaglandin E_2_ (PGE_2_), assessed by EIA, as well as interleukin (IL)-10 and transforming growth factor (TGF)-β, assessed by ELISA, in nonpreconditioned (AD-MSC) and EPA-preconditioned MSCs (AD-MSC-EPA). Data are presented as median ± interquartile range of three independent experiments

Nonpreconditioned and EPA-preconditioned AD-MSCs similarly displayed the characteristic morphology, adherence to plastic culture flasks, and expression of MSC-specific surface markers (negative for CD31, CD45, and MHCII; positive for CD29, CD49e, and CD44) (Fig. 1). Additionally, EPA preconditioning did not affect cell survival in comparison to nonpreconditioned AD-MSCs (Fig. 2). Both nonpreconditioned and EPA-preconditioned AD-MSCs also demonstrated formation of exosomes and microvesicles on MSC surfaces (Fig. 3). EPA-stimulated AD-MSCs, compared with nonpreconditioned AD-MSCs, presented increased levels of RvD1, PGE2, IL-10, and TGF-β (Fig. 4).

### EPA-preconditioned AD-MSCs led to further improvements in clinical score and survival rate than nonpreconditioned AD-MSCs

**Fig. 5:**
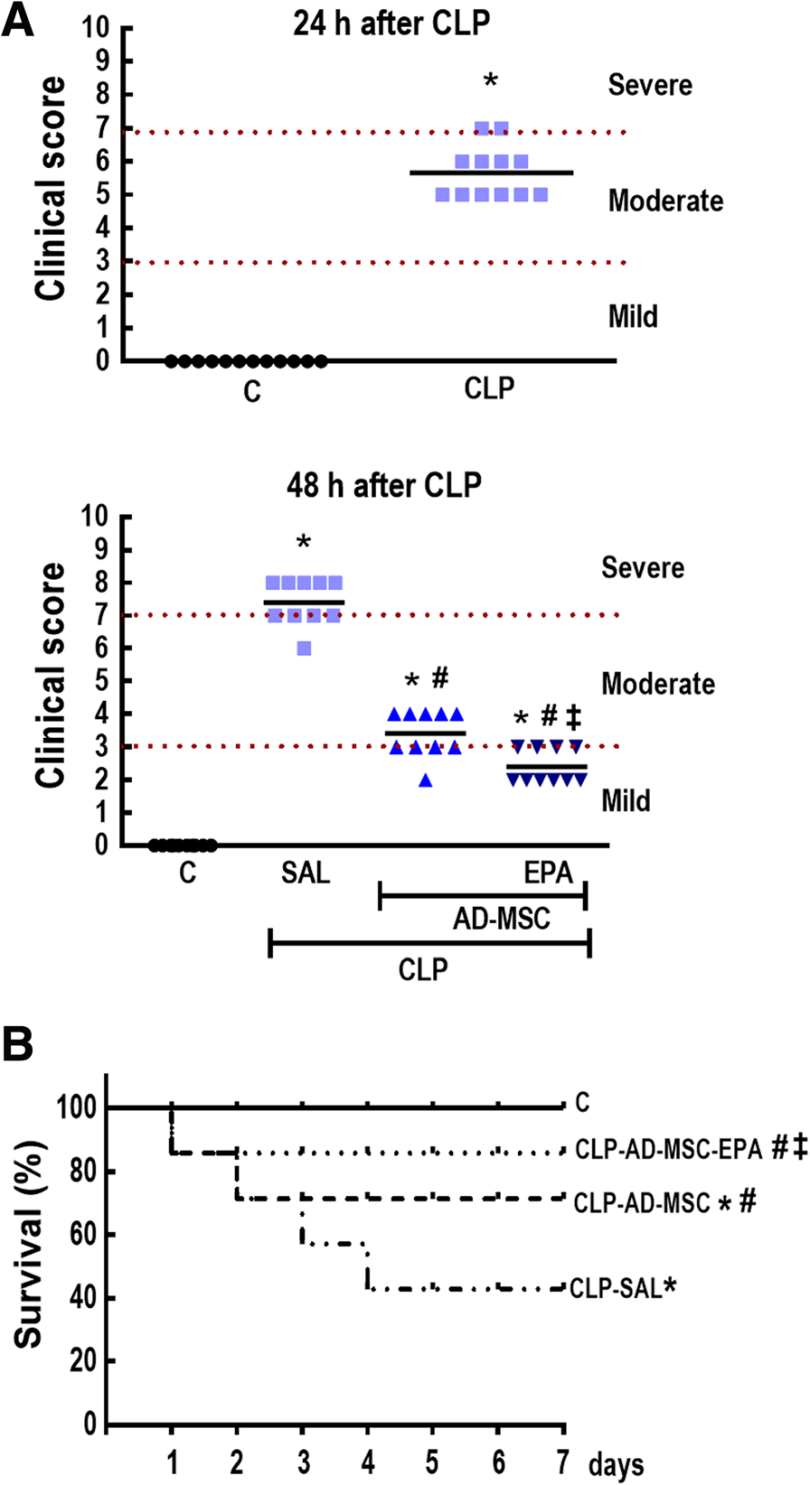
Sepsis severity score (**a**) and survival rate (**b**). **a** Clinical score evaluated 24 h and 48 h after cecal ligation and puncture (CLP) and sham surgery (C). **b** Kaplan–Meier survival curves after sham surgery (C) or cecal ligation and puncture (CLP)-induced sepsis over 7 days. At 24 h, the CLP group was further randomized to receive saline (0.05 mL, SAL), adipose tissue-derived mesenchymal stromal cells (AD-MSC; 10^5^ cells) (nonpreconditioned), or preconditioned with eicosapentaenoic acid (AD-MSC-EPA; 10^5^ cells). *Significantly different from the C group (*P* < 0.05). ^#^Significantly different from the CLP-SAL group (*P* < 0.05). ^‡^Significantly different from the CLP-AD-MSC group (*P* < 0.05)

On day 1, the CLP group demonstrated clinical features of moderate disease, while no abnormal features were observed in C mice (Fig. 5a). On day 2, the CLP-SAL group demonstrated clinical features of severe disease, while C mice continued to show no abnormal features. Compared to CLP-SAL, clinical score was significantly improved to levels consistent with moderate and mild disease in mice receiving nonpreconditioned and EPA-preconditioned AD-MSCs, respectively.

On day 7, the survival rate of C animals was 100%, versus 43% in the CLP-SAL group (Fig. 5b). Administration of nonpreconditioned AD-MSCs improved survival in CLP animals to 71%, while administration of EPA-preconditioned AD-MSCs improved survival to 86%.

### EPA-preconditioned AD-MSCs led to a further improvement in lung mechanics than nonpreconditioned AD-MSCs

**Fig. 6:**
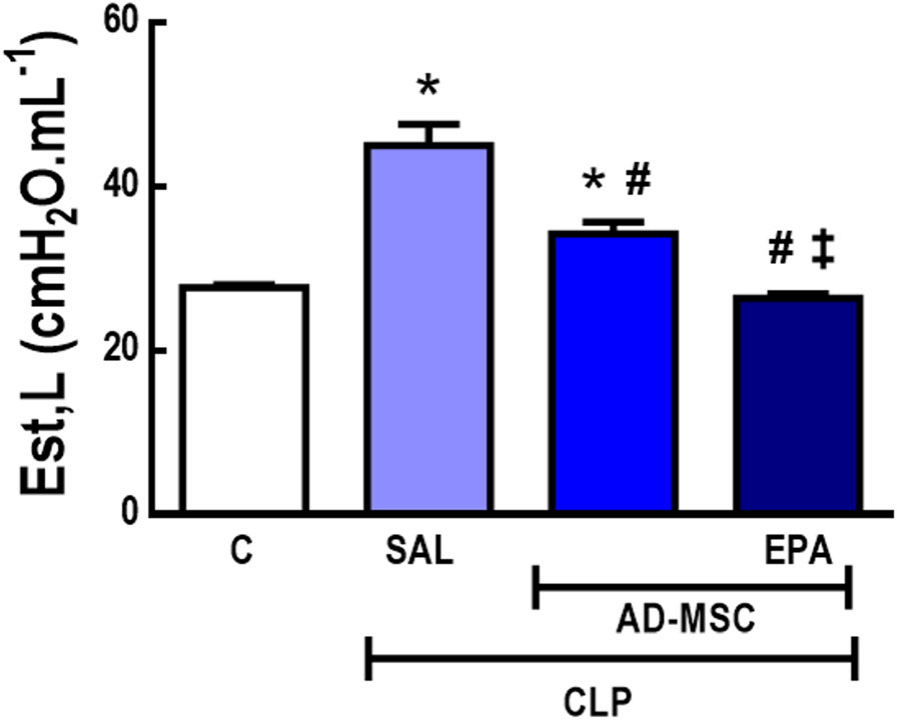
Static lung elastance. Sepsis was induced by cecal ligation and puncture (CLP), while sham-operated animals were used as control (C). At 24 h, the CLP group was further randomized to receive saline (0.05 mL, SAL), adipose tissue-derived mesenchymal stromal cells (AD-MSC; 10^5^ cells) (nonpreconditioned), or preconditioned with eicosapentaenoic acid (AD-MSC-EPA; 10^5^ cells). Values are mean + SD of eight animals/group. *Significantly different from the C group (*P* < 0.05). ^#^Significantly different from the CLP-SAL group (*P* < 0.05). ^‡^Significantly different from the CLP-AD-MSC group (*P* < 0.05)

The CLP-SAL group demonstrated higher Est,L compared to C mice (63%) (Fig. 6). Nonpreconditioned and EPA-preconditioned AD-MSCs reduced Est,L; however, EPA-preconditioned AD-MSCs were more effective than nonpreconditioned AD-MSCs at reducing Est,L to C-comparable levels.

### EPA-preconditioned AD-MSCs were more effective at reducing alveolar collapse, interstitial edema, inflammation, and collagen fiber content than nonpreconditioned AD-MSCs

**Fig. 7:**
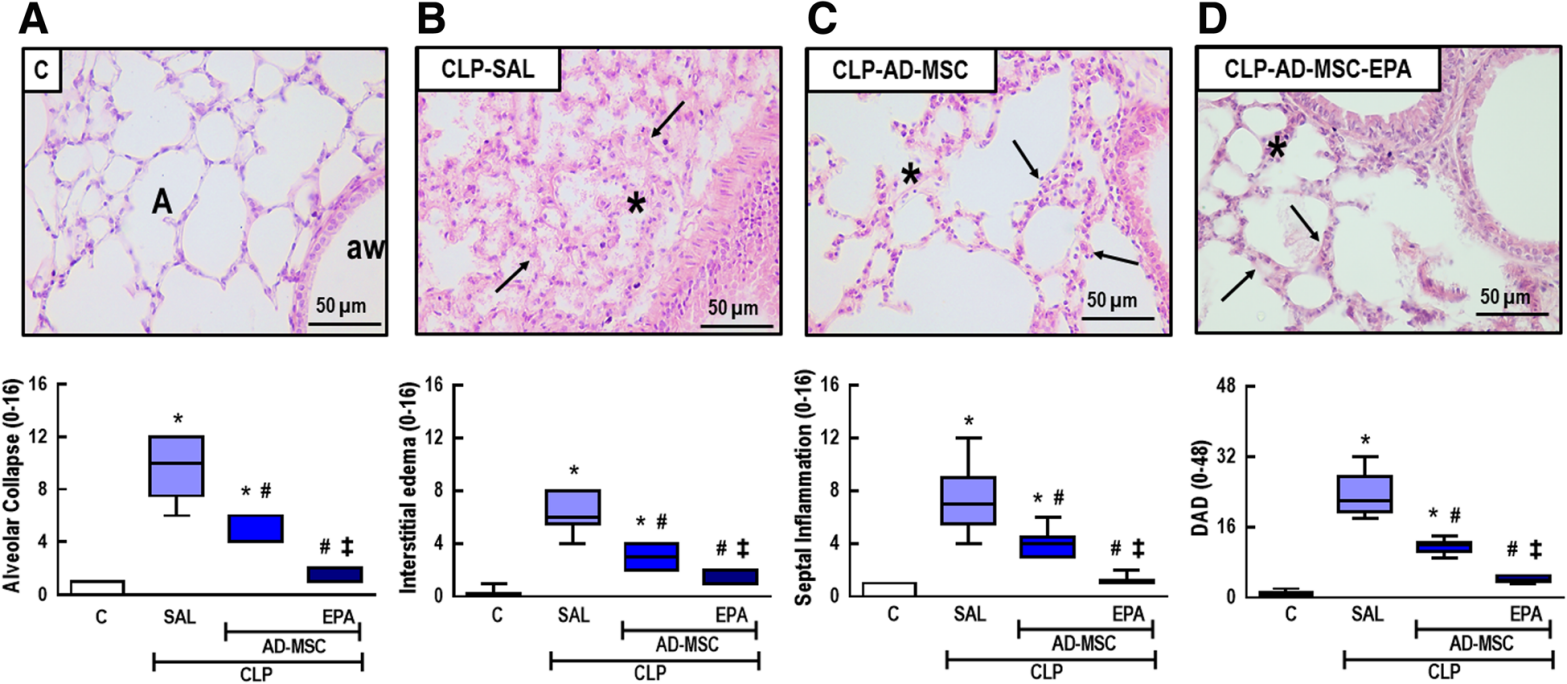
Upper panels: Representative photomicrographs of lung parenchyma stained with hematoxylin-eosin. (**a**) in the C group, lung architecture presents normal alveoli (A) and airways (AW); (**b**) in the CLP-SAL group, lung damage was observed, with areas of alveolar collapse (asterisk) and alveolar septal inflammation (arrows); (**c**) administration of nonpreconditioned AD-MSCs (CLP-AD-MSC) attenuated alveolar collapse (asterisk) and alveolar septal inflammation (arrows); and (**d**) administration of EPA-preconditioned AD-MSCs (CLP-AD-MSC-EPA) led to a further reduction in alveolar collapse (asterisk) and alveolar septal inflammation (arrows). Lower panels: Cumulative diffuse alveolar damage (DAD) score representing injury from alveolar collapse, interstitial edema, and septal inflammation. Values are given as medians (interquartile ranges) of 8 animals in each group. *Significantly different from C group (*P* <0.05). #Significantly different from CLP-SAL group (*P* <0.05). ‡Significantly different from CLP-AD-MSC group (*P* <0.05)

Representative photomicrographs of lung parenchyma showed increased areas of alveolar collapse and alveolar septal inflammation (Fig. 7, upper panels). AD-MSCs attenuated alveolar collapse and alveolar septal inflammation, whereas administration of EPA-preconditioned AD-MSCs led to a further reduction in these parameters. DAD score, which represents the severity of alveolar collapse, interstitial edema, and alveolar septal inflammation, was increased in CLP-SAL compared to the C group, due to statistically significant increases in these three parameters (Fig. 7, lower panels). Nonpreconditioned AD-MSCs led to a significant reduction in alveolar collapse, interstitial edema, and alveolar septal inflammation, resulting in a decrease in DAD score; however, EPA-preconditioned AD-MSCs were even more effective at reducing these abnormalities to C-comparable levels.

The CLP-SAL group demonstrated greater fraction area of mononuclear cells and neutrophils in lung tissue, and higher collagen fiber content compared to C mice (Table 1). Nonpreconditioned and EPA-preconditioned AD-MSCs reduced all these parameters, but EPA-preconditioned AD-MSCs were even more effective at reducing fraction area of neutrophil cell count, and collagen fiber content.

**Table 1:** Lung morphometry

Groups	Mononuclear cells (%)	Neutrophils (%)	Collagen fibers (%)
C	33.6 ± 0.7	2.5 ± 0.3	0.20 ± 0.02
CLP			
SAL	48.9 ± 1.4*	19.2 ± 1.9*	0.45 ± 0.06*
AD-MSC	39.2 ± 1.5*^,#^	7.3 ± 0.8*^,#^	0.35 ± 0.05*^,#^
AD-MSC-EPA	37.3 ± 1.3*^,#^	3.5 ± 0.9*^,#,‡^	0.26 ± 0.03*^,#,‡^

Fraction area of mononuclear cells, neutrophils, and collagen fibers in alveolar septa. Control (C) mice were subjected to an abdominal incision alone, while animals were subjected to cecal ligation and puncture (CLP) for induction of polymicrobial sepsis. After 24 h, the CLP group was further randomized to receive saline (0.05 mL, SAL) or adipose tissue-derived mesenchymal stromal cells (MSC) maintained under regular conditions (AD-MSC; 10^5^ cells) or preconditioned with eicosapentaenoic acid (AD-MSC-EPA; 10^5^ cells). *n* = 8/group. *Significantly different from the C group (*P* < 0.05). ^#^Significantly different from the CLP-SAL group (*P* < 0.05). ^‡^Significantly different from the CLP-AD-MSC group (*P* < 0.05)

### Nonpreconditioned and EPA-preconditioned AD-MSCs reduced protein levels of inflammatory and fibrotic biomarkers in lung tissues, but EPA-preconditioned AD-MSCs further reduced IL-1β and KC as well as increased VEGF levels

**Fig. 8:**
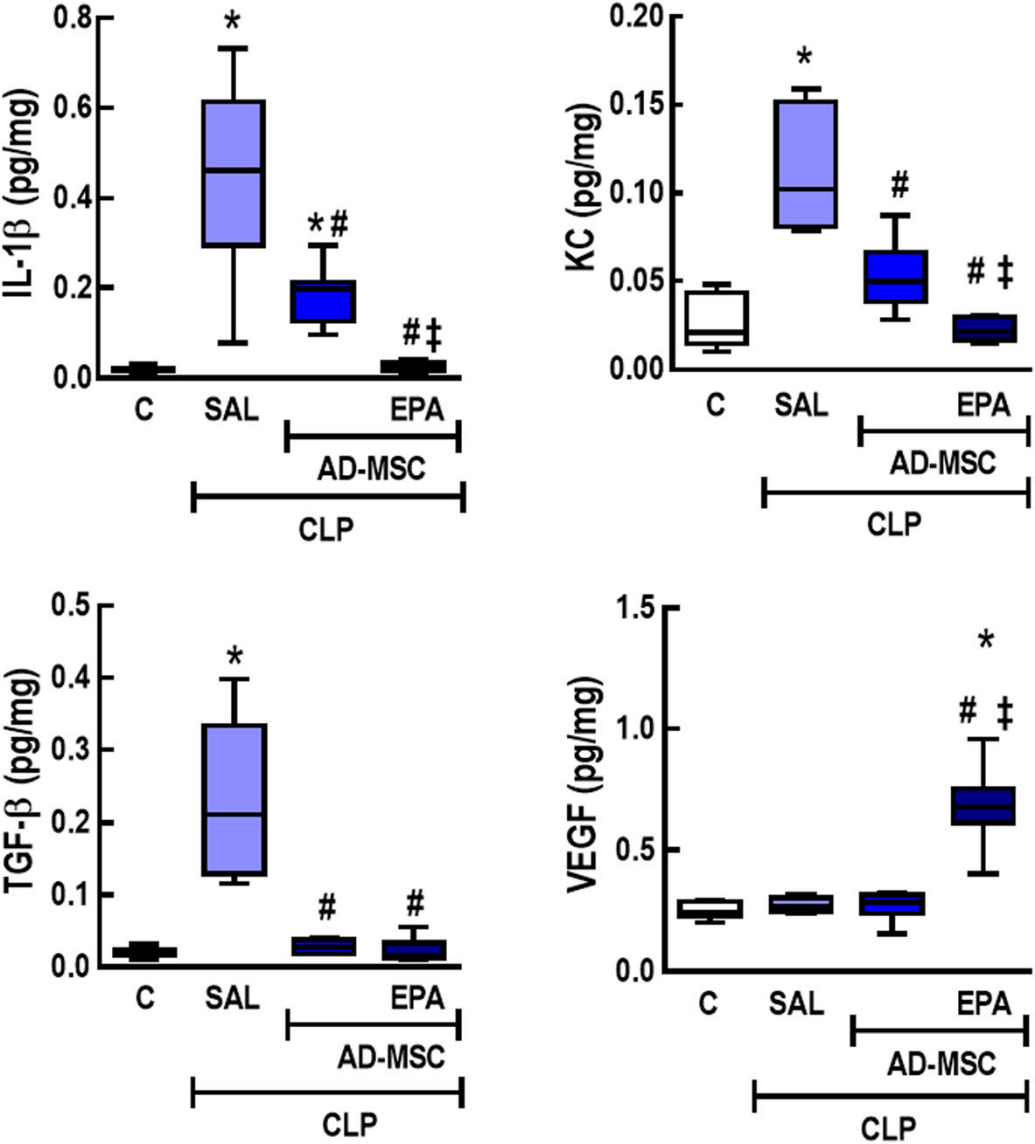
Protein levels of mediators in lung tissue homogenate. Protein levels of interleukin (IL)-1β, keratinocyte chemoattractant (KC), transforming growth factor (TGF)-β, and vascular endothelial growth factor (VEGF) in lung tissue homogenate. CLP animals underwent cecal ligation and puncture, while C mice underwent sham surgery. At 24 h, the CLP group was further randomized to receive saline (0.05 mL, SAL), adipose tissue-derived mesenchymal stromal cells (AD-MSC; 10^5^ cells) (nonpreconditioned), or preconditioned with eicosapentaenoic acid (AD-MSC-EPA; 10^5^ cells). *n* = 8/group. *Significantly different from the C group (*P* < 0.05). ^#^Significantly different from the CLP-SAL group (*P* < 0.05). ^‡^Significantly different from the CLP-AD-MSC group (*P* < 0.05)

Protein levels of pro-inflammatory (IL-1β, KC) and pro-fibrotic (TGF-β) mediators were increased in lung tissue homogenates from CLP-SAL compared to C mice (Fig. 8). Nonpreconditioned and EPA-preconditioned AD-MSCs reduced protein levels of IL-1β and KC, but only EPA-preconditioned AD-MSCs decreased these mediators to C-comparable levels. Nonpreconditioned and EPA-preconditioned AD-MSCs similarly reduced the level of TGF-β. Furthermore, C, CLP-SAL, and CLP-AD-MSC animals demonstrated similar VEGF protein levels; however, this parameter was significantly higher in CLP mice receiving EPA-preconditioned AD-MSCs. No differences were observed in IL-10 protein levels among experimental groups (data not shown).

### EPA-preconditioned AD-MSCs were more effective than nonpreconditioned AD-MSCs to reduce total cells, monocytes, and neutrophils in the blood

**Fig. 9:**
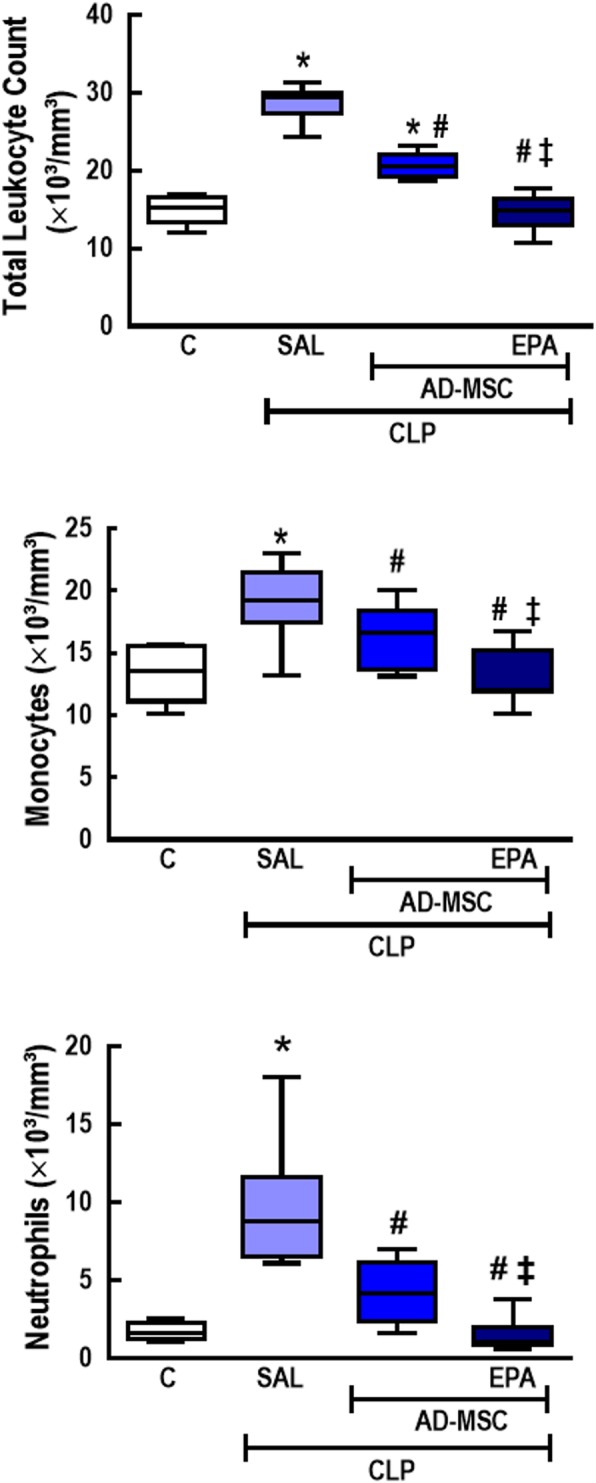
Total and differential cell counts in blood. Total cell, monocyte, and neutrophil counts in blood. CLP animals underwent cecal ligation and puncture, while C mice underwent sham surgery. At 24 h, the CLP group was further randomized to receive saline (0.05 mL, SAL), adipose tissue-derived mesenchymal stromal cells (AD-MSC; 10^5^ cells) (nonpreconditioned), or preconditioned with eicosapentaenoic acid (AD-MSC-EPA; 10^5^ cells). *n* = 8/group. *Significantly different from the C group (*P* < 0.05). ^#^Significantly different from the CLP-SAL group (*P* < 0.05). ^‡^Significantly different from the CLP-AD-MSC group (*P* < 0.05)

CLP-SAL animals exhibited an increase in total and differential cellularity in the blood compared to C mice (Fig. 9). Both nonpreconditioned and EPA-preconditioned AD-MSCs reduced all these cell counts, but EPA-preconditioned AD-MSCs were even more effective and reduced total cells, monocytes, and neutrophils in the blood to C-comparable levels.

### Only EPA-preconditioned AD-MSCs enhanced alveolar macrophage capacity for phagocytosis

Alveolar macrophages from C and CLP-SAL groups exhibited similar phagocytosis capacity (Additional file 2: Figure S2). Co-culture of alveolar macrophages from CLP mice with AD-MSCs led to enhanced phagocytosis capacity in alveolar macrophages only when AD-MSCs were preconditioned with EPA.

### EPA-preconditioned AD-MSCs were more effective at reducing abnormalities in distal organs than nonpreconditioned AD-MSCs

**Fig. 10:**
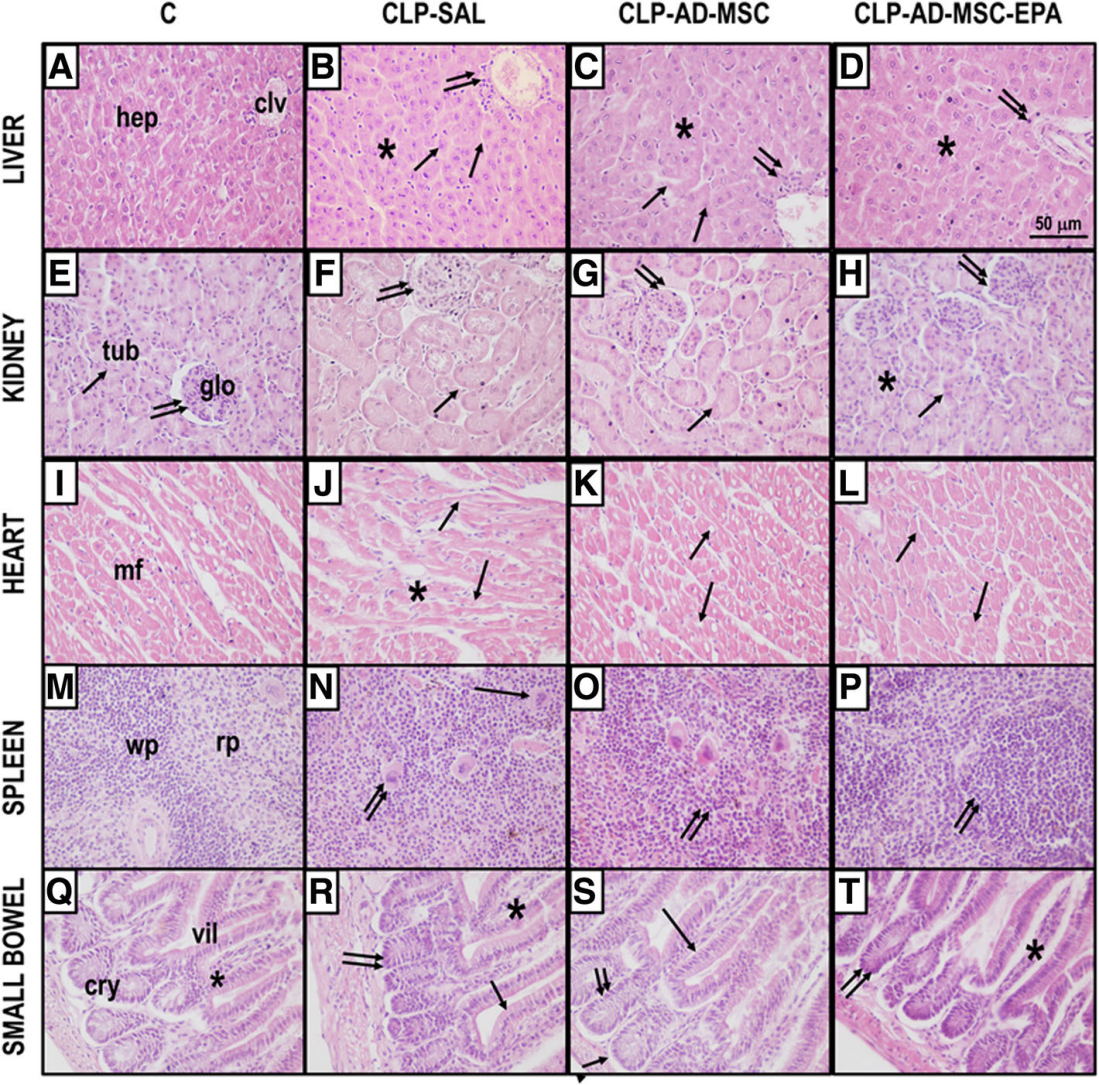
Representative photomicrographs of distal organs. Photomicrographs of slices from the liver (**a**–**d**), kidney (**e**–**h**), heart (**i**–**l**), spleen (**m**–**p**), and small bowel (**q**–**t**) stained with hematoxylin and eosin. **a** Normal liver architecture with intact hepatocytes (hep) surrounding the centrolobular vein (clv); **b** in the CLP-SAL group, liver damage was observed, with hepatocyte disarrangement (asterisk), hydropic degeneration of hepatocytes (single arrow), and an increased number of Kupffer cells (double arrows); **c** administration of nonpreconditioned AD-MSCs attenuated the liver architecture disarrangement (asterisk), hydropic degeneration of hepatocytes (single arrow), and number of Kupffer cells (double arrows); **d** administration of EPA-preconditioned AD-MSCs increased the number of regenerated hepatocytes (asterisk) and decreased the number of Kupffer cells (double arrows). **e** Normal kidney architecture with intact glomeruli (glo) and renal tubules (tub); **f** in the CLP-SAL group, kidney damage was characterized by acute tubular necrosis (single arrow) with glomerular damage (double arrows); **g** administration of nonpreconditioned AD-MSCs attenuated both the acute tubular necrosis (single arrow) and glomerular damage (double arrows); **h** administration of EPA-preconditioned AD-MSCs increased the number of regenerated renal tubules (asterisk) and further attenuated acute tubular necrosis (single arrow) and glomerular damage (double arrows). **i** Normal heart architecture with intact muscle fibers (mf); **j** in the CLP-SAL group, heart damage was observed, with interstitial edema (asterisk) and microvacuolization of muscle fibers (arrows); **k** administration of nonpreconditioned AD-MSCs attenuated the interstitial edema and microvacuolization (arrows); **l** administration of EPA-preconditioned AD-MSCs decreased the interstitial edema and restored muscle fibers (arrows). **m** Normal spleen architecture with white pulp (wp) and red pulp (rp); **n** in the CLP-SAL group, spleen damage was observed, with activation of lymphocytes in white pulp, increased number of megakaryocytes in red pulp (single arrows), and augmented number of lymphoblasts and lymphocytes in white pulp (double arrows); **o** administration of nonpreconditioned AD-MSCs maintained the activation of lymphocytes (double arrows) and decreased number of megakaryocytes in red pulp; **p** administration of EPA-preconditioned AD-MSCs decreased the reactivity of lymphoid follicles (double arrows) and the number of megakaryocytes in red pulp. **q** Normal architecture of small bowel with preserved villi (vil), lamina propria (asterisk), and crypt of Lieberkuhn (cry); **r** in the CLP-SAL group, small-bowel damage was observed, characterized by villi enlargement (single arrow), edema of lamina propria (asterisk), and increased number of enteroblasts in crypts of Lieberkuhn (double arrows); **s** administration of nonpreconditioned MSCs mitigated the villi enlargement (single arrow) and reduced number of enteroblasts in crypts of Lieberkuhn (double arrows); **t** administration of EPA-preconditioned AD-MSCs restored normal small bowel architecture mitigated edema of the lamina propria (asterisk) and led to a further reduction in the number of enteroblasts in crypts of Lieberkuhn (double arrows). Sepsis was induced by cecal ligation and puncture (CLP), while sham-operated animals were used as control (C). Twenty-four hours after surgery, the CLP group received saline (0.05 mL, SAL), adipose tissue-derived mesenchymal stromal cells (AD-MSC; 10^5^ cells) (nonpreconditioned), or preconditioned with eicosapentaenoic acid (AD-MSC-EPA; 10^5^ cells)

The CLP-SAL group demonstrated several morphological abnormalities in distal organs (the liver, kidney, heart, spleen, and small bowel) compared to C mice (Table 2 and Fig. 10). Both nonpreconditioned and EPA-preconditioned AD-MSCs reduced such abnormalities; however, therapeutic effects on the liver, kidney, heart, spleen, and small bowel were even more pronounced in mice receiving EPA-stimulated AD-MSCs.

**Table 2:** Semiquantitative analysis of distal organ damage

Pathologic findings	Groups			
	C	CLP-SAL	CLP-AD-MSC	CLP-AD-MSC-EPA
Liver				
Hepatocyte disarrangement	1 (0.5–1)	9 (8.5–10.5)*	8 (7–8.5)*	2 (2–3)*^,#,‡^
Kupffer cell hyperplasia	1 (1–1)	9 (9–9)*	6 (6–6)*^,#^	4 (3–4)*^,#,‡^
Hydropic degeneration	1 (1–1)	6 (5–7.5)*	3 (3–4.5)*^,#^	2 (2–3)*^,#^
Kidney				
Tubular acute necrosis	0 (0–0.5)	12 (10.5–12)*	6 (5–6)*^,#^	2 (2–3)*^,#,‡^
Heart				
Disarrangement of cardiac myocyte architecture	1 (0.5–1)	6 (5–6)*	4 (3–4)*^,#^	2 (2–3)*^,#,‡^
Interstitial edema	1 (0.5–1)	6 (6–7.5)*	2 (2–2)*^,#^	2 (1.5–2)*^,#^
Microvacuolization	1 (1–1)	9 (7.5–9)*	2 (1.5–2)*^,#^	2 (1.5–2)*^,#^
Spleen				
Increased number of megakaryocytes	1 (0.5–1)	12 (10.5–12)*	6 (5–6)*^,#^	2 (2–3)*^,#,‡^
Increased number of lymphocytes	1 (1–1.5)	8 (7–8)*	6 (5–6)*^,#^	2 (2–2)*^,#,‡^
Small bowel				
Villi architecture disorganization	1 (0.5–1)	12 (10–12)*	6 (5–6)*^,#^	2 (2–3)*^,#,‡^
Edema of lamina propria	0 (0–0.5)	9 (8.5–9)*	4 (4–5)*^,#^	4 (3–4)*^,#^
Number of enteroblasts (crypt cells)	0 (0–0.5)	6 (6–7)*	4 (4–5)*^,#^	4 (3–4)*^,#^

Values are median (interquartile range) of eight mice in each group. A semiquantitative scoring system based on severity and extension of injury was used to assess the degree of distal organ damage. Severity was graded as follows: 1 = normal tissue, 2 = mild injury, 3 = moderate injury, and 4 = severe injury. Extension of injury was graded as follows: 0 = normal tissue, 1 = damage to 1–25% of total tissue examined, 2 = damage to 26–50% of total tissue examined, 3 = damage to 51–75% of total tissue examined, and 4 = damage to 76–100% of total tissue examined. The final score is obtained by multiplying severity and extension of injury in each tissue examined. Scoring was done independently by two different investigators (J.D.S. and V.L.C.). Control (C) mice were subjected to an abdominal incision alone, while animals were subjected to cecal ligation and puncture (CLP) to induce polymicrobial sepsis. After 24 h, the CLP group was further randomized to receive saline (0.05 mL, SAL) or adipose tissue-derived mesenchymal stromal cells maintained under regular conditions (MSC; 10^5^ cells) or preconditioned with eicosapentaenoic acid (AD-MSC-EPA; 10^5^ cells). *Significantly different from the C group (*P* < 0.05). ^#^Significantly different from the CLP-SAL group (*P* < 0.05). ^‡^Significantly different from CLP-AD-MSC group (*P* < 0.05)

### EPA preconditioning did not affect AD-MSC distribution to tissues

One hour after administration, ^99m^Tc-labeled AD-MSCs were present in several tissues, with higher prevalence in the lungs and kidneys. EPA preconditioning elicited no significant differences in AD-MSC biodistribution (Additional file 3: Figure S3).

## Discussion

In the model of CLP-induced sepsis used herein, EPA-preconditioned AD-MSCs yielded greater therapeutic benefits in comparison to nonpreconditioned AD-MSCs by further reducing lung mechanics and histological abnormalities, inflammation, collagen fiber content, and morphological alterations in distal organs, while increasing VEGF levels in lung tissue. Additionally, lower disease severity scores and a higher survival rate were observed in CLP mice receiving EPA-preconditioned AD-MSCs.

CLP is one of the most relevant experimental models for polymicrobial sepsis research, since it resembles several features of human disease, including multiple organ dysfunction and high mortality [20, 26]. In this study, AD-MSCs were administered 24 h after CLP surgery to consider the time course of lung and distal organ injury, in contrast to previous studies in which MSCs were administered few hours after injury [7, 8, 27, 28]. Although the bone marrow is the most commonly used source of MSCs, the cells used herein were collected from adipose tissue, as it is estimated to contain a higher number of MSCs and, in the clinical setting, is readily accessible by liposuction [29, 30]. Furthermore, in a previous study of experimental acute respiratory distress syndrome, bone marrow-derived and adipose tissue-derived MSCs similarly improved lung function, reduced lung inflammation and remodeling, and mitigated liver and kidney injury [5]. In initial clinical trials, both bone marrow-derived and adipose tissue-derived MSCs appeared to be safe and feasible for the treatment of critically ill patients [12–14], although further optimizations seem to be required to maximize therapeutic benefits.

The mechanisms by which MSCs exert their therapeutic actions remain incompletely elucidated, but paracrine secretion of trophic factors is considered a key underlying mechanism [5, 15]. Under regular conditions, MSCs can secrete or induce other cells to secrete several paracrine factors; however, recent studies have attempted to stimulate MSCs with certain agents prior to administration in order to enhance secretion of factors that could potentially result in further beneficial effects [6, 15–17]. In this context, EPA can modify the fatty acid composition of membranes in several cell types, as well as serve as a substrate for the cyclooxygenase and lipoxygenase enzymes, thus altering cell signaling and gene expression and reducing production of arachidonic acid-derived mediators [18, 19]. Although EPA preconditioning did not affect AD-MSC morphology, surface markers, in vitro survival, or in vivo biodistribution in the present study, we observed that EPA actively increased secretion of anti-inflammatory and pro-resolution mediators by AD-MSCs, including resolvin D_1_, PGE_2_, IL-10, and TGF-β, as in a previous study by our group that used bone marrow-derived MSCs in allergic asthma [17].

The acute respiratory distress syndrome is a common and serious complication observed in septic patients [1]. Pathogen- and damage-associated molecular patterns trigger a complex pro-inflammatory response, leading to increased secretion of a wide range of pro-inflammatory mediators, including IL-1β and KC, which results in intense cellular infiltration into the lungs and loss of epithelial integrity [20, 31]. Nonpreconditioned AD-MSC administration led to reduction in protein levels of such mediators, significantly reducing neutrophil cell count in lung tissue, while improving lung mechanics. Notably, EPA-preconditioned AD-MSCs further improved some (but not all) of these endpoints, including alveolar neutrophilia in lung tissue, interstitial edema, and alveolar septal thickening. EPA preconditioning can mobilize the production of lipid-derived pro-resolution mediators, such as resolvins and protectins, that mitigate the production of pro-inflammatory mediators and prevent neutrophilic infiltration by inhibiting transendothelial migration [32, 33].

Endothelial injury facilitates pulmonary edema formation and contributes to remodeling of the alveolar-capillary membrane [20, 31]. Both nonpreconditioned and EPA-preconditioned AD-MSCs were able to reduce TGF-β levels, interstitial edema, and collagen fiber content in the lung; however, EPA-preconditioned AD-MSCs were more effective at reducing alveolar collapse, interstitial edema, and lung fibrosis, while also increasing VEGF levels in lung tissue. The effects of cell-based therapy on VEGF levels in experimental lung injury have been conflicting; some studies have demonstrated increased expression [34, 35], whereas others indicated a reduction [5, 36]. Such discrepancies can be attributed to differences in experimental model and severity (CLP vs. endotoxin), cell source and type (mononuclear cells vs. bone marrow-derived or adipose tissue-derived MSCs), administration route (intratracheal vs. intravenous), and timing of therapy and analysis. In agreement with our current findings, another study indicated that MSCs increased VEGF levels in lung tissue, correlating with recovery of vascular endothelial cadherin, protection against endothelial apoptosis, and reduction of lung permeability in experimental lung injury. All these beneficial effects were significantly reduced by VEGF knockdown [37]. Additionally, EPA preconditioning can enhance MSC production of PGE_2_ [17], which can act as a negative regulator of collagen secretion by promoting more efficient wound healing and re-epithelization [38–40]. PGE_2_ also inhibits inflammation and neutrophil influx [39, 41] and stimulates macrophage polarization into the M2 profile rather than M1 [17, 28, 42]. In this line, our results indicate that only macrophages exposed to EPA-preconditioned AD-MSCs presented an increase in phagocytosis capacity, which can result in a more efficient bacterial clearance.

Sepsis-induced systemic inflammation leads to morphological and functional abnormalities in multiple organs, resulting in clinical deterioration over time and, ultimately, death. MSC therapy has been demonstrated to protect the lungs and distal organs from damage in experimental models of sepsis by exerting antioxidative, anti-apoptotic, and anti-inflammatory effects [5, 28, 43, 44]. In this line, both nonpreconditioned and EPA-preconditioned AD-MSCs reduced blood leukocyte counts and abnormalities in distal organs in the present study. Although no differences in AD-MSC distribution across tissues were observed, EPA-preconditioned AD-MSCs were greatly effective at reducing number of circulating inflammatory cells as well as abnormalities in the lungs and other organs, correlating with further improvements in the clinical score and survival of septic mice.

## Conclusion

EPA-preconditioned AD-MSCs increased secretion of pro-resolution and anti-inflammatory mediators (RvD_1_, PGE_2_, IL-10, and TGF-β) by these cells. In CLP-induced sepsis, this was associated with further reductions in lung and distal organ damage, thus resulting in greater improvements in clinical score and higher survival rate, compared to therapy with nonpreconditioned AD-MSCs. This may be a promising therapeutic approach to improve outcome in septic patients.

## Additional files

Additional file 1: **Figure S1.** Schematic flowchart of the study design and timeline. (DOCX 39 kb)

Additional file 2: **Figure S2.** Phagocytosis assay. (DOCX 58 kb)

Additional file 3: **Figure S3.** AD-MSC biodistribution. (DOCX 165 kb)

## Data Availability

The datasets during and/or analyzed during the current study are available from the corresponding author on reasonable request.

## Abbreviation

AD-MSC: Adipose tissue-derived mesenchymal stromal cells
CLP: Cecal ligation and puncture
DAD: Diffuse alveolar damage
EPA: Eicosapentaenoic acid
Est,L: Static lung elastance
IL: Interleukin
KC: Keratinocyte chemoattractant
MSC: Mesenchymal stromal cell
PGE2: Prostaglandin E2
Tc: Technetium
TGF-β: Transforming growth factor
VEGF: Vascular endothelial growth factor

## References

[1] Gotts JE, Matthay MA (2016). Sepsis: pathophysiology and clinical management. BMJ..

[2] Duggal A, Ganapathy A, Ratnapalan M, Adhikari NK (2015). Pharmacological treatments for acute respiratory distress syndrome: systematic review. Minerva Anestesiol.

[3] Antunes MA, Abreu SC, Cruz F, Teixeira AC, Lopes-Pacheco M, Bandeira E (2014). Effects of different mesenchymal stromal cell sources and delivery routes in experimental emphysema. Respir Res.

[4] Morrison TJ, Jackson MV, Cunningham EK, Kissenpgenning A, McAuley DF, O’Kane CM, Krasnodembskaya AD (2017). Mesenchymal stromal cells modulate macrophages in clinically relevant lung injury models by extracellular vesicles mitochondrial transfer. Am J Respir Crit Care Med.

[5] Silva JD, Lopes-Pacheco M, Paz AHR, Cruz FF, Melo EB, de Oliveira M (2018). Mesenchymal stem cells from bone marrow, adipose tissue, and lung tissue differentially mitigate lung and distal organ damage in experimental acute respiratory distress syndrome. Crit Care Med.

[6] Abreu SC, Xisto DG, de Oliveira TB, Blanco NG, de Castro LL, Kitoko JZ (2019). Serum from asthmatic mice potentiates the therapeutic effects of mesenchymal stromal cells in experimental allergic asthma. Stem Cells Transl Med.

[7] Krasnodembskaya A, Samarani G, Song Y, Zhuo H, Su X, Lee JW (2012). Human mesenchymal stem cells reduce mortality and bacteremia in gram-negative sepsis in mice in part by enhancing the phagocytic activity of blood monocytes. Am J Physiol Lung Cell Mol Physiol..

[8] Alcayaga-Miranda F, Cuenca J, Martin A, Contreras L, Figeuroa FE, Khoury M (2015). Combination therapy of menstrual derived mesenchymal stem cells and antibiotics ameliorates survival in sepsis. Stem Cell Res Ther.

[9] Chen HH, Lin KC, Wallace CG, Chen YT, Yang CC, Leu S (2014). Additional benefit of combined therapy with melatonin and apoptotic adipose-derived mesenchymal stem cell against sepsis-induced kidney injury. J Pineal Res.

[10] Condor JM, Rodrigues CE, Sousa Moreira R, Canale D, Volpini RA, Shimizu MH (2016). Treatment with human Wharton’s jelly-derived mesenchymal stem cells attenuated sepsis-induced kidney injury, liver, injury and endothelial dysfunction. Stem Cell Trans Med.

[11] Devaney J, Horie S, Masterson C, Elliman S, Barry F, O’Brien T (2015). Mesenchymal stromal cells decrease the severity of acute lung injury induced by *E. coli* in the rat. Thorax.

[12] Zheng G, Huang L, Tong H, Shu Q, Hu Y, Ge M (2014). Treatment of acute respiratory distress syndrome with allogeneic adipose-derived mesenchymal stem cells: a randomized, placebo-controlled pilot study. Respir Res.

[13] Wilson JG, Liu KD, Zhuo H, Caballero L, McMillan M (2015). Mesenchymal stem (stromal) cells for treatment of ARDS: a phase 1 clinical trial. Lancet Respir Med.

[14] Matthay MA, Calfee CS, Zhuo H, Thompson BT, Wilson JG, Levitt JE (2019). Treatment with allogeneic mesenchymal stromal cells for moderate to severe acute respiratory distress syndrome (START study): a randomized phased 2a safety trial. Lancet Respir Med.

[15] Silva LHA, Antunes MA, Dos Santos CC, Weiss DJ, Cruz FF, Rocco PRM (2018). Strategies to improve the therapeutic effects of mesenchymal stromal cells in respiratory diseases. Stem Cell Res Ther.

[16] Lan YW, Choo KB, Chen CM, Hung TH, Chen YB, Hsieh CH (2015). Hypoxia-preconditioned mesenchymal stem cells attenuate bleomycin-induced pulmonary fibrosis. Stem Cell Res Ther.

[17] Abreu SC, Lopes-Pacheco M, da Silva AL, Xisto DG, de Oliveira TB, Kitoko JZ (2018). Eicosapentaenoic acid enhances the effects of mesenchymal stromal cell therapy in experimental allergic asthma. Front Immunol.

[18] Calder PC (2013). Omega-3 polyunsaturated fatty acids and inflammatory processes: nutrition or pharmacology?. Br J Clin Pharmacol.

[19] Freitas HR, Isaac AR, Malcher-Lopes R, Diaz BL, Trevenzoli IH, De Melo Reis RA (2018). Polyunsatured fatty acids and endocannabinoids in health and disease. Nutr Neurosci.

[20] Hubbard William J, Choudhry Mashkoor, Schwacha Martin G, Kerby Jeffrey D, Rue Loring W, Bland Kirby I, Chaudry Irshad H (2005). CECAL LIGATION AND PUNCTURE. Shock.

[21] Zhang X, Goncalves R, Mosser DM (2008). The isolation and characterization of murine macrophages. Curr Protoc Immunol.

[22] Bates JH, Ludwig MS, Sly PD, Brown K, Martin JG, Fredberg JJ (1988). Interrupter resistance elucidated by alveolar pressure measurement in open-chest normal dogs. J Appl Physiol (1985).

[23] Hsia CC, Hyde DM, Ochs M, Weibel ER (2010). An official research policy statement of the American Thoracic Society/European Respiratory Society: standards for quantitative assessment of lung structure. Am J Respir Crit Care Med.

[24] Silva JD, Paredes BD, Araújo IM, Lopes-Pacheco M, Oliveira MV, Suhett GD (2014). Effects of bone marrow-derived mononuclear cells from healthy or acute respiratory distress syndrome donors on recipient lung-injured mice. Crit Care Med.

[25] Lopes-Pacheco M, Ventura TG, de Oliveira HD, Monção-Ribeiro LC, Gutfilen B, dos Souza SA (2014). Infusion of bone marrow mononuclear cells reduces lung fibrosis but not inflammation in the late stage of murine silicosis. PLoS One.

[26] Ruiz S, Vardon-Boues F, Merlet-Dupuy V, Conil JM, Buléon M, Fourcade I (2016). Sepsis modeling in mice: ligation length is a major severity factor in cecal ligation and puncture. Intensive Care Med Exp.

[27] Mei SH, Haitsma JJ, dos Santos CC, Deng Y, Lai PF, Slutsky AS (2010). Mesenchymal stem cells reduce inflammation while enhancing bacterial clearance and improving survival in sepsis. Am J Respir Crit Care Med.

[28] Nemeth K, Leelahavanichkyl A, Yuen PS, Mayer B, Parmelee A, Doi K (2009). Bone marrow stromal cells attenuate sepsis via prostaglandin E(2)-dependent reprograming of host macrophages to increase their interleukin-10 production. Nat Med.

[29] McIntyre LA, Moher D, Fergusson DA, Sullivan KJ, Mei SH, Lalu M (2016). Efficacy of mesenchymal stromal cell therapy for acute lung injury in preclinical animal models: a systemic review. PLoS One.

[30] Peng L, Jia Z, Yin X, Zhang X, Liu Y, Chen P (2008). Comparative analysis of mesenchymal stem cells from bone marrow, cartilage and adipose tissue. Stem Cells Dev.

[31] Hotchkiss RS, Moldawer LL, Opal SM, Reinhart K, Turnbull IR, Vincent JL (2016). Sepsis and septic shock. Nat Rev Dis Primers.

[32] Schwab JM, Chiang N, Arita M, Serhan CN (2007). Resolvin E1 and protectin D1 activate inflammation-resolution programmes. Nature..

[33] Sun YP, Oh SF, Uddin J, Yang R, Gotlinger K, Campbell E (2007). Resolvin D1 and its aspirin-triggered 17R epimer. Stereochamical assignments, anti-inflammatory properties, and enzymatic inactivation. J Biol Chem.

[34] Araújo IM, Abreu SC, Maron-Gutierrez T, Cruz F, Fujisaki L, Carreira H (2010). Bone marrow-derived mononuclear cell therapy in experimental pulmonary and extrapulmonary acute lung injury. Crit Care Med.

[35] Chang YS, Ahn SY, Jeon HB, Sung DK, Kim ES, Sung SI (2014). Critical role of vascular endothelial growth factor secreted by mesenchymal stem cells in hyperoxic lung injury. Am J Respir Cell Mol Biol.

[36] Lee SH, Jang AS, Kim YE, Cha JY, Kim TH, Jung S (2010). Modulation of cytokine and nitric oxide by mesenchymal stem cell transfer in lung injury/fibrosis. Respir Res.

[37] Yang Y, Hu S, Xu X, Li J, Liu A, Han J, Liu S, Liu L, Qiu H. The vascular endothelial growth factors-expressing character of mesenchymal stem cells plays a positive role in treatment of acute lung injury in vivo. Mediat Inflamm. 2016; 2016:2347938. 10.1155/2016/2347938 PMC4895047 27313398

[38] Moore BB, Ballinger MN, White ES, Green ME, Herrygers AB, Wilke CA (2005). Bleomycin-induced E prostanoid receptor changes alter fibroblast responses to prostaglandin E2. J Immunol.

[39] Dackor RT, Cheng J, Voltz JW, Card JW, Ferguson CD, Garrett RC (2011). Prostaglandin E2 protects murine lung from bleomycin-induced pulmonary fibrosis and lung dysfunction. Am J Physiol Lung Cell Mol Physiol.

[40] Miyoshi H, VanDussen KL, Malvin NP, Ryu SH, Wang Y, Sonnek NM (2017). Prostaglandin E2 promotes intestinal repair through an adaptive cellular response of the epithelium. EMBO J.

[41] Birrell MA, Maher SA, Dekkak B, Jones V, Wong S, Brook P, Belvisi MG (2015). Anti-inflammatory of PGE2 in the lung: role of the EP4 receptor subtype. Thorax..

[42] Vasandan AB, Jahnavi S, Shashank C, Prasad P, Kumar A, Prasanna SJ (2016). Human mesenchymal stem cells program macrophage plasticity by altering their metabolic status via a PGE_2_-dependent mechanism. Sci Rep.

[43] Mohammadzadeh M, Halabian R, Gharehbaghian A, Amirizadeh N, Jahaniam-Najafabadi A, Roushandeh AM, Roudkenar MH (2012). Nrf-2 overexpression in mesenchymal stem cells reduced oxidative stress-induced apoptosis and cytotoxicity. Cell Stress Chaperones.

[44] Lee FY, Chen KH, Wallace CG, Sung PH, Sheu JJ, Cheng SY (2017). Xenogeneic human umbilical cord-derived mesenchymal stem cells reduce mortality in rats with acute respiratory distress syndrome complicated by sepsis. Oncotarget..

