# Supplementary material for: Eicosapentaenoic acid potentiates the therapeutic effects of adipose tissue-derived mesenchymal stromal cells on lung and distal organ injury in experimental sepsis
Source: Stem Cell Res Ther. 2019 Aug 23;10:264. doi: 10.1186/s13287-019-1365-z (PMC6708232; doi:10.1186/s13287-019-1365-z)
Supplement: Supplementary file 1 — Figure S1. Schematic flowchart of the study design and timeline. (DOCX 39 kb) [file 13287_2019_1365_MOESM1_ESM.docx]

**Additional File 1**
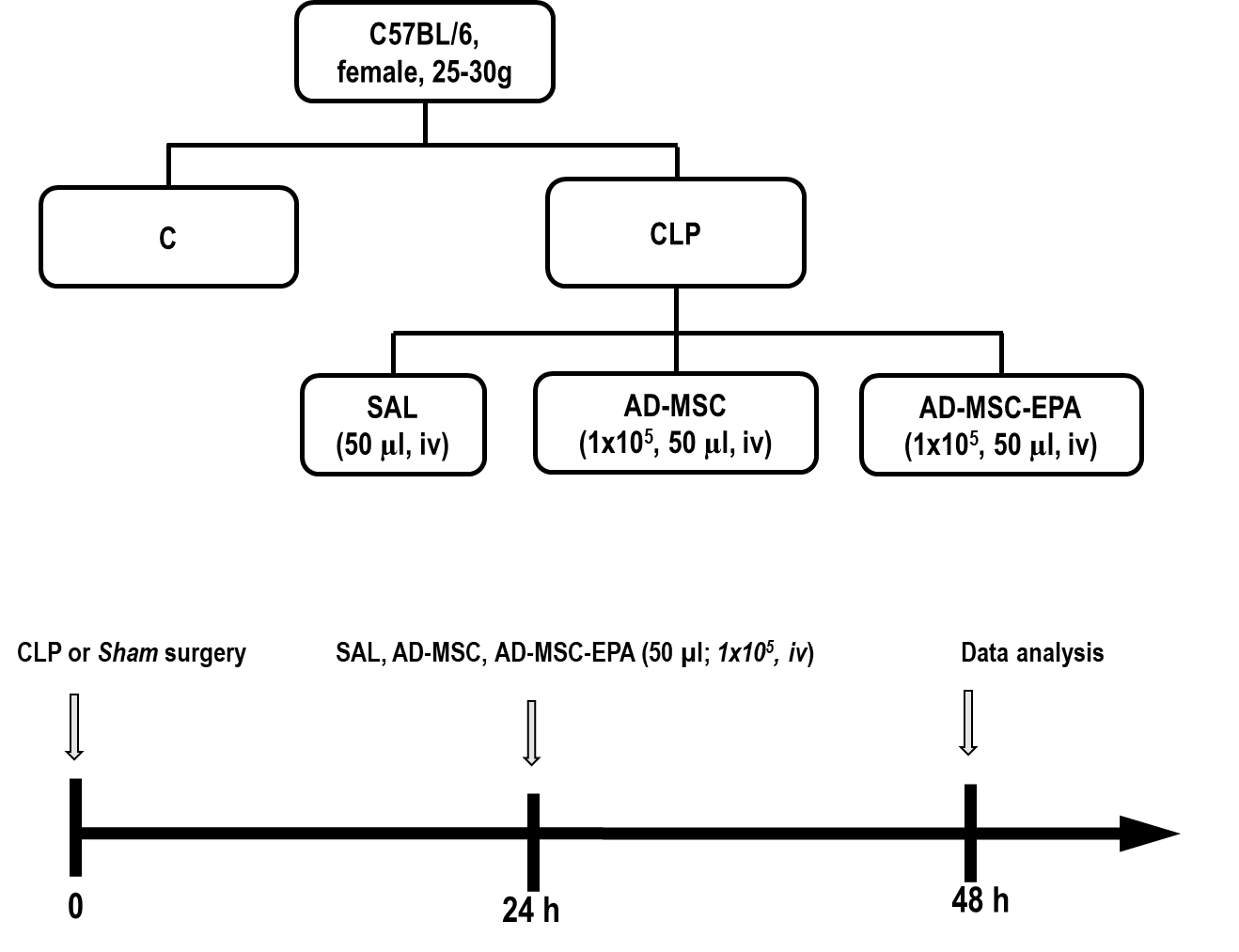


**Figure S1 - Schematic flowchart of the study design and timeline.** Sepsis was induced by cecal ligation and puncture (CLP), while sham-operated animals were used as control (C). At 24 h, the CLP group was further randomized to receive saline (0.05 mL, SAL) or adipose tissue-derived mesenchymal stromal cells (AD-MSC; 10^5^ cells) (non-preconditioned) or preconditioned with eicosapentaenoic acid (AD-MSC-EPA; 10^5^ cells).
