# Supplementary material for: Eicosapentaenoic acid potentiates the therapeutic effects of adipose tissue-derived mesenchymal stromal cells on lung and distal organ injury in experimental sepsis
Source: Stem Cell Res Ther. 2019 Aug 23;10:264. doi: 10.1186/s13287-019-1365-z (PMC6708232; doi:10.1186/s13287-019-1365-z)
Supplement: Supplementary file 2 — Figure S2. Phagocytosis assay. (DOCX 58 kb) [file 13287_2019_1365_MOESM2_ESM.docx]

**Additional File 2**

**Figure S2 – Phagocytosis assay.** Quantification of fluorescence by absorbance spectrum. Sepsis was induced by cecal ligation and puncture (CLP), while sham-operated animals were used as control (C). Twenty-four hours after surgery, CLP animals were treated with intravenous infusion of sterile saline (SAL), or adipose tissue-derived mesenchymal stromal cells (AD-MSC; 10^5^ cells) (non-preconditioned) or preconditioned with eicosapentaenoic acid (AD-MSC-EPA; 10^5^ cells). Data are presented as box plots (median and interquartile range) of 5 mice in each group. *Significantly different from C group (P<0.05). **^#^**Significantly different from CLP-SAL group (P<0.05).
