# Supplementary material for: Eicosapentaenoic acid potentiates the therapeutic effects of adipose tissue-derived mesenchymal stromal cells on lung and distal organ injury in experimental sepsis
Source: Stem Cell Res Ther. 2019 Aug 23;10:264. doi: 10.1186/s13287-019-1365-z (PMC6708232; doi:10.1186/s13287-019-1365-z)
Supplement: Supplementary file 3 — Figure S3. AD-MSC biodistribution. (DOCX 165 kb) [file 13287_2019_1365_MOESM3_ESM.docx]

**Additional File 3**


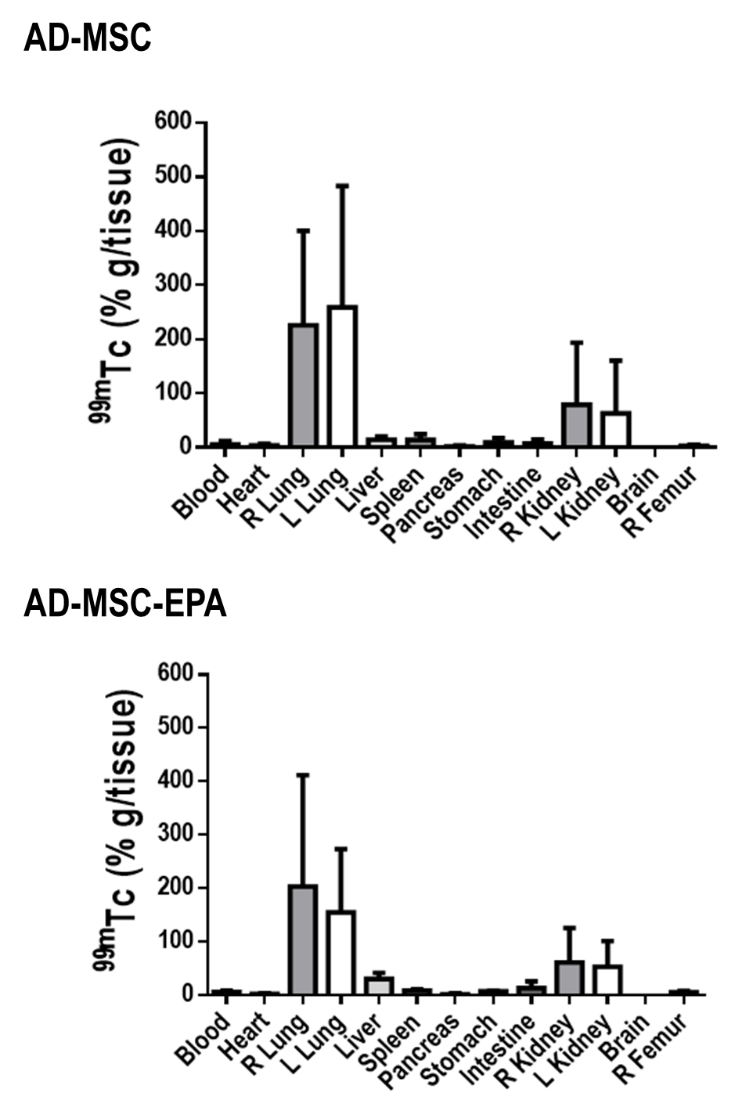


**Figure S3 – AD-MSC biodistribution.** Quantification of tissue scintigraphy 1 hour after administration of nonpreconditioned (A) (AD-MSC) or EPA-preconditioned AD-MSCs (B) labelled with ^99m^Tc. Sepsis was induced by cecal ligation and puncture (CLP) surgery. Twenty-four after surgery, CLP animals were treated with adipose tissue derived mesenchymal stromal cells (AD-MSC) (nonpreconditioned) or AD-MSCs preconditioned with eicosapentaenoic acid for 6 hours (AD-MSC-EPA).
